# Supplementary material for: Evidence for the utility of quantum computing before fault tolerance
Source: Nature. 2023 Jun 14;618(7965):500–5. doi: 10.1038/s41586-023-06096-3 (PMC10266970; doi:10.1038/s41586-023-06096-3)
Supplement: Supplementary file 1 — Supplementary Sections 1–10, including Supplementary Figs. 1–13, Supplementary Table 1 and Supplementary References. [file 41586_2023_6096_MOESM1_ESM.pdf]

nature portfolio

https://doi.org/10.1038/s41586-023-06096-3

Supplementary information

Evidence for the utility of quantum computing before fault tolerance

In the format provided by the authors and unedited

Supplementary Information: Evidence for the utility of quantum computing before fault tolerance

Youngseok Kim*,[1] Andrew Eddins*,[2] Sajant Anand,[3] Ken Xuan Wei,[1] Ewout van den Berg,[1] Sami Rosenblatt,[1] Hasan Nayfeh,[1] Yantao Wu,[3,4] Michael Zaletel,[3,5] Kristan Temme,[1] and Abhinav Kandala[1]

[1] *IBM Quantum, IBM T.J. Watson Research Center, Yorktown Heights, NY 10598, USA*
[2] *IBM Quantum, MIT-IBM Watson AI lab, Cambridge MA, 02142, USA*
[3] *Dept. of Physics, University of California, Berkeley, CA 94720, USA*
[4] *RIKEN iTHEMS, Wako, Saitama 351-0198, Japan*
[5] *Material Science Division, Lawrence Berkeley National Laboratory, Berkeley, California 94720, USA*

(Dated: April 5, 2023)

I. DEVICE PROPERTIES

This supplementary material includes device data from *ibm_kyiv*, a 127 qubit processor, and *ibm_ithaca*, a 65 qubit processor. Although the two devices differ in their qubit numbers and readout properties, other attributes such as coherence times and gate error rates are comparable. While we produce the data for main-text experiments on *ibm_kyiv*, we used *ibm_ithaca* to develop calibration and data-processing strategy and investigate the impact of device non-idealities on error mitigation outcomes.

Our device consists of qubits arranged with a heavy-hexagonal lattice topology [S1]. Single qubit gates are calibrated to have 49.78 ns wide Gaussian envelope pulse with DRAG correction [S2]. The device properties are summarized in Table 1. Two qubit gates were defined using the echoed cross-resonant (CR) pulse sequence [S3–S6]. As no qubit has more than three neighbors, the two qubit gates can be separated into three depth-1 layers, see Fig. S1(a) for *ibm_kyiv* and Fig. S1(b) for *ibm_ithaca*. Each layer was set to have a uniform two qubit length of 611.56 ns for *ibm_kyiv* and 512 ns for *ibm_ithaca*. The two qubit gates were calibrated simultaneously and Fig. S1(c, d) summarizes the distribution of two qubit randomized benchmarking results that are performed simultaneously per layer. We use the identical randomized benchmarking sequences within each layer to mimic the situation where the same length of two qubit gates are applied in parallel within each layer. We emphasize that the gate error is in general worse than benchmark results from isolated two qubit pairs, as (i) most of the gates are slower compared to the case when each individual gates are optimized separately, as we choose to use a uniform gate length for each layer, and (ii) benchmarking isolated pairs cannot capture spectator associated errors where driving a nearest-neighbor qubit (spectator) could induce errors on the cross-resonant gate operation [S7].

The readout length for *ibm_kyiv* is 782 ns for average $\chi/2\pi = 1.11 \pm 0.20$ MHz and $\kappa/2\pi = 3.80 \pm 1.09$ MHz. Meanwhile, *ibm_ithaca* has average $\chi/2\pi = 1.46 \pm 0.51$ MHz and $\kappa/2\pi = 0.28 \pm 0.04$ MHz, which is lower than the value in *ibm_kyiv*. Therefore, the readout length is tuned up to be longer as 4.55 μs. To initialize each qubit, we first measure the qubit state, then apply a π pulse conditioned on $|1\rangle$. We repeat this conditional reset procedure twice as an initialization protocol. The resultant readout fidelity is illustrated in Fig. S1(e, f).

	ibm_kyiv (127Q)				*ibm_ithaca* (65Q)			
	median	mean	min	max	median	mean	min	max
f_{01} (GHz)	4.61	4.62 ± 0.11	4.34	4.96	4.73	4.74 ± 0.10	4.54	4.93
$f_{01} - f_{12}$ (MHz)	311.07	310.77 ± 11.48	280.56	356.92	333.36	333.99 ± 5.61	316.04	355.56
T_1 (μs)	287.87	293.39 ± 84.78	85.30	567.55	183.54	180.52 ± 43.44	77.92	278.08
T_2 (μs)	127.49	156.73 ± 109.47	16.18	456.02	183.70	182.88 ± 91.05	19.27	407.15

TABLE S1. **Summary of single qubit properties on *ibm_kyiv* and *ibm_ithaca*.** Reported T_1, T_2 were obtained from mean values of daily measurements over a 7-day period.

II. CLASSICAL PROCESSING PROCEDURES

In this section, we discuss the detailed protocol of the error mitigation procedure.

A. Circuit randomization

The idea behind ZNE is to evaluate observable expectation values for circuits with different noise-gain levels and extrapolate from these values the desired zero-noise error-mitigated observable. In our approach, the circuits are assumed to consist of layers of noisy two-qubit gates, interleaved with noise-free or low-noise single-qubit gates. The noise of each layer ℓ is shaped using Pauli twirls and modeled as sparse Pauli-Lindblad noise channels Λ_ℓ. We amplify the noise to the desired noise-gain level $G \geq 1$ by applying the scaled noise channel Λ_ℓ^{G-1} before each layer ℓ. Moreover, to enable readout-error mitigation, we apply Pauli-X twirling of the measurements at the end of the circuit [S8]. The implementation of the twirls and noise amplification channels leverages linearity of the expectation value and amounts to sampling circuits from a distribution that implements the desired operations in expectation. The sampling of a single circuit instance proceeds as follows. First, note that each of the noise amplification channels $\Lambda_\ell^{G_1}$ represent a (highly structured) n-qubit Pauli channel $\sum_{i=0}^{4^n-1} p_i P_i \cdot P_i^\dagger$. The coefficients $\{p_i\}$ of any Pauli channel form a probability distribution, and we therefore replace the channel by a single Pauli P_i operator sampled according to this distribution, using the efficient sampling procedure described in Methods section. Second, we implement the Pauli-twirled version of the noisy layer by applying a Pauli P_i operator, sampled uniformly at random, followed by

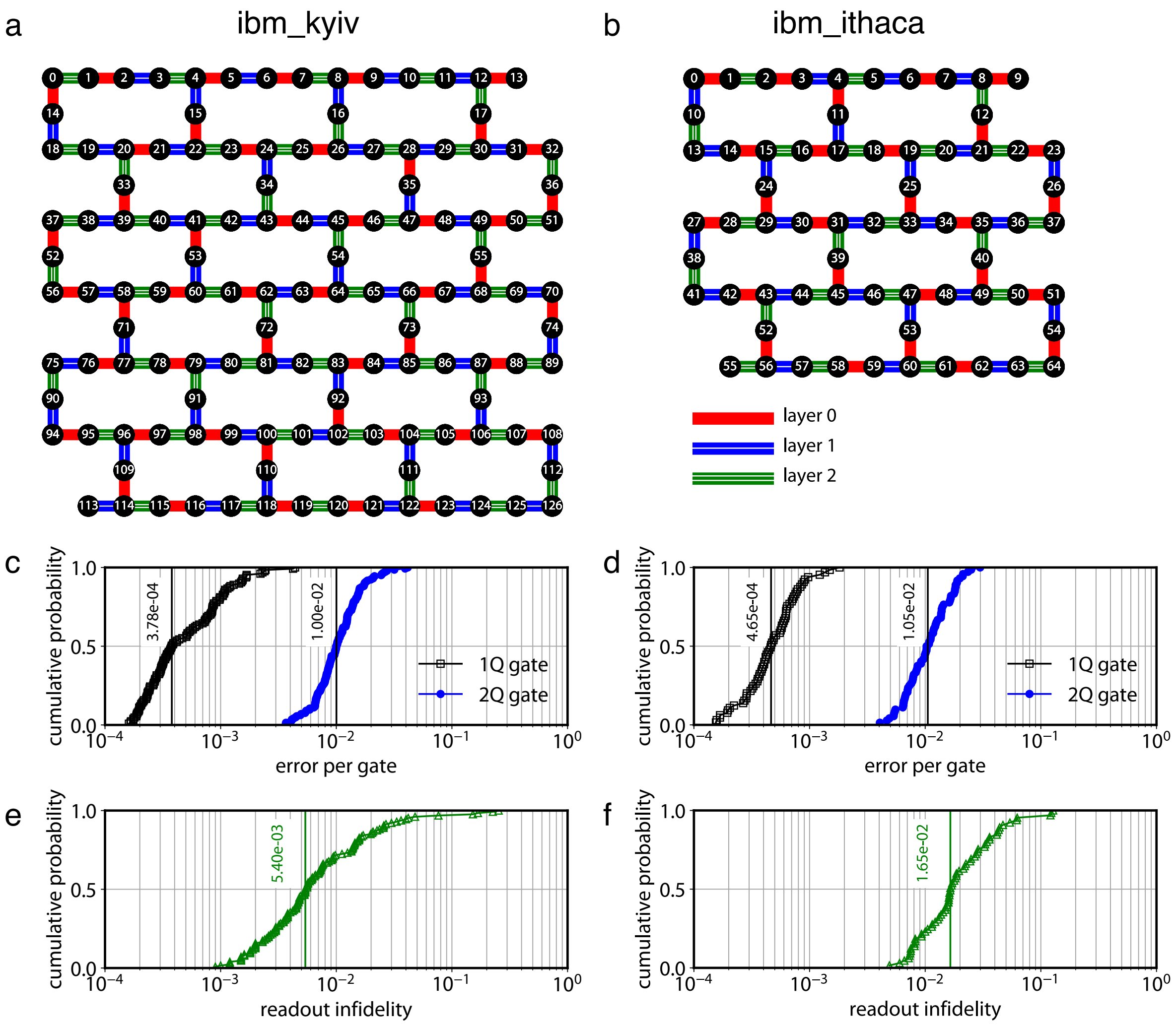

FIG. S1. **Summary of single/two qubit gate and readout fidelity distribution on** ***ibm_kyiv*** **and** ***ibm_ithaca*****.** All the two qubit gates are batched into three layers for (a) *ibm_kyiv* and (b) *ibm_ithaca*. (c) Single/two qubit error per gate (EPG) distribution for *ibm_kyiv*. Median value is indicated in the plot. The two qubit gates within the same layer are calibrated and evaluated simultaneously. Mean value of single qubit EPG is $6.75 \times 10^{-4} \pm 6.80 \times 10^{-4}$ and mean value of two qubit EPG is $1.15 \times 10^{-2} \pm 0.61 \times 10^{-2}$. (d) Single/two qubit error per gate (EPG) distribution for *ibm_ithaca*. Median value is indicated in the plot. The two qubit gates within the same layer are calibrated and evaluated simultaneously. Mean value of single qubit EPG is $5.44 \times 10^{-4} \pm 3.17 \times 10^{-4}$ and mean value of two qubit EPG is $1.17 \times 10^{-2} \pm 0.54 \times 10^{-2}$. (e) Readout fidelity distribution for *ibm_kyiv*. Median value is indicated in the plot, and the mean value is $1.53 \times 10^{-2} \pm 3.57 \times 10^{-2}$. (f) Readout fidelity distribution for *ibm_ithaca*. Median value is indicated in the plot, and the mean value is $2.56 \times 10^{-2} \pm 2.55 \times 10^{-2}$.

the noisy layer, and the conjugation of P_i by the ideal (noise-free) layer. Third, we precede all final measurements by a Pauli X or I operator, sampled uniformly at random, and classically implement the corresponding bit-flips to all measurements. All other gates are left unchanged. It is easily seen that each part implements the desired operation in expectation, and it then follows directly from independence of the components that we sample our circuits from the desired distribution.

B. Data accumulation and extrapolation

We illustrate the data accumulation and extrapolation protocol for the mitigated estimation of a single observable $\langle O = X_2 \rangle$ on a 65-qubit circuit consisting of four Trotter steps with $\theta_h = 0$, with states prepared and measured in the Pauli-X basis, as illustrated in Fig. S3(a). Extrapolation is done at noise-gain levels $G \in \{1, 1.1, 1.34, 1.58\}$,

corresponding to tilted Chebyshev nodes [S9]. We prepare $N_b = 54$ batches of 100 randomized circuit instances for each of the four different gain levels and acquire 64 samples per circuit by sequentially running all batches on IBM's 65-qubit processor *ibm_ithaca*. Following readout-error mitigation [S8] on the data we obtain the time-series data shown in Fig. S2(a). The time-series shows large fidelity degradations for circuit instances 3500 to 4500, corresponding to batches 35–45. The temporal locality of these aberrations suggests they are caused by transient degradations in the device performance, such as the resonant interaction of qubits with two-level systems (TLSs) [S10, S11], which we further analyze in Sec. III. To identify such outliers, we use thresholding based on the median absolute deviation (MAD). That means that, given the time-series estimates $[O] = [\langle O\rangle_1, \langle O\rangle_2, \ldots, \langle O\rangle_{N_b}]$, we identify batch i as an outlier whenever

$$|\langle O\rangle_i - \mathrm{median}([O])| > 2\bar{\sigma}, \quad (1)$$

where $\bar{\sigma} = k\cdot\mathrm{MAD}$ describes the deviation of the observable with $\mathrm{MAD} = \mathrm{median}(|[O]-\mathrm{median}([O])|)$ and $k = 1.4826$ is chosen assuming normally distributed data. Following this procedure, we identify seven batches, indicated as vertical lines in Fig. S2(a), as outliers and exclude these batches from all further processing. We obtain the final observable estimate by averaging the $\langle O\rangle_i$ values of the remaining batches. The cumulative mean of $\langle O\rangle_i$ over the selected data stabilizes after around 3000 accumulated circuit instances, as shown in Fig. S2(b).

Given the observable estimates for the different gain levels, we can perform extrapolation to the zero-noise limit. Figure S2(c) plots the mitigated observable value obtained using linear (empty symbol) and exponential (filled blue symbol) least-square fits. The estimate closest to the ideal value of 1 is obtained using the exponential fit. We determine error bars on the final mitigated observables by means of bootstrapping. That means the data are resampled and reprocessed to obtain a set of estimates that can then be used to estimate confidence intervals. Random sampling occurs twice in the experiment: once when generating random circuit instances, and again when repeatedly sampling each circuit instance on the quantum hardware. We therefore first resample circuits, uniformly at random with replacement, from the pool of circuit instances. We then resample shots from the pool of shots for each of the sampled circuit instance. In our experiment we generate 100 mitigated expectation values this way for and plot the median and $\pm 68\%$ quantiles in Fig. S2(c). Fig. S2(d) illustrates the corresponding plot for increasing amounts of data. While the extrapolated value converges near 1, the corresponding confidence interval obtained from bootstrap also decreases for more data accumulation.

Using the same batch data, we repeat the procedure outlined in Fig. S2(a–d) for all 65 weight-one Pauli-Z observables O_q. Figure S2(e) plots the distribution of $\langle O_q\rangle$ over the 100 bootstrap instances along with the global magnetization

$$M_O = (1/65)\sum_q \langle O_q\rangle, \quad (2)$$

for the different fitting methods. We found that exponential extrapolation can become unstable for large circuit depths when the expectation values cluster around zero, even for small noise-gain levels. Similar unstable behavior can occur for high-order polynomial extrapolation in the form of Runge's phenomenon [S12], where overfitting leads to large fluctuations outside the sampled domain. As a way of regulating these instabilities we reject results in which the uncertainty of the extrapolated estimate, taken as the square root of the diagonal element of the covariance matrix returned by the fit routine, exceeds a threshold value of 0.5. Based on this criterion we successively downgrade the estimation from exponential fit to linear fit, and from linear fit to the unmitigated result obtained for noise-gain level $G = 1$. This pragmatic solution was performed on both experimental and bootstrapped results independently, and we leave further improvements to this approach for future studies. Quadratic fits were found to produce larger variance and larger error compared to exponential extrapolation (see for instance Fig. S2(e)), and were therefore not used for any of the results in the main text.

C. Beyond optimizing mitigation protocols

The two error mitigation protocols ZNE and PEC set out to improve expectation values within the allotted coherence time of the device. For these protocols the hardware noise sets a constant coherence limit in both the depth and the number of qubits. A rough estimate [S13] for some generic device noise parameter λ indicates that one requires the product $nL\lambda$, for n - qubits and circuit depth L, to be small. As such, these mitigation protocols are not expected to increase the circuit depth beyond what is permitted by the hardware constants. Recently derived information theoretic bounds, that are independent of the specific error mitigation protocols, provide formal support for this picture [S14–S16]. This indicates that going forward beyond the optimization of the protocols taking into account the actually relevant circuit volume [S17], the central contribution for increased circuit volumes will be the improvement in hardware noise.

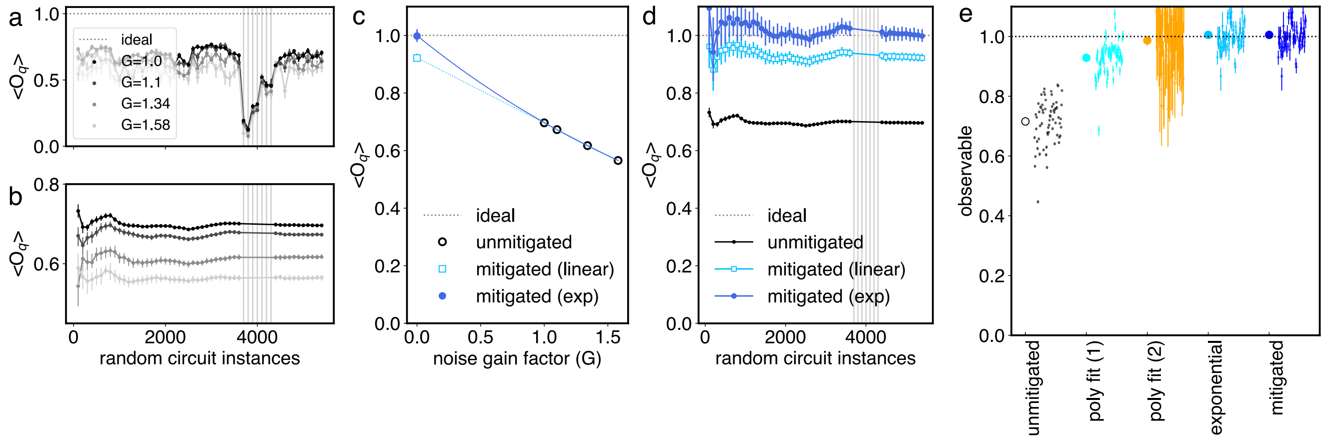

FIG. S2. **Data processing procedures.** As an example, we illustrate processing steps for estimating $\langle O_q = X_2 \rangle$ after 4 Trotter steps of the circuit in Fig. S3(a) using data from *ibm_ithaca* at noise amplification factors $G = 1, 1.1, 1.34, 1.58$. (a) Each point represents an expectation value obtained from a batch of 100 random circuit realizations for Pauli/readout twirling and random Pauli gate insertion for noise amplification. Error bars indicate 68% confidence interval for all panels, obtained from bootstrapping 100 configurations; some are smaller than markers. Vertical lines indicate outliers identified from Eq. 1. (b) The cumulative averages converge after a few thousands of random circuit instances. Vertical lines emphasize excluded outliers identified in (a). (c) Error mitigation (ZNE) is performed using four gain factors (three in the main text). First order polynomial fit and exponential order extrapolation (exp) are plotted along with the unmitigated experimental results. (d) Error mitigation is performed as a function of accumulated data. The ideal value is 1 for this observable, indicated as a dotted horizontal line. The exponential extrapolation outperforms the first order polynomial fit. (e) The unmitigated and mitigated observables on the 65 qubits are plotted to show the estimated observable distribution across 65 qubit device.

D. Trotter experiments on *ibm_ithaca*

As a demonstration of reproducibility of our methods, we measure error mitigated observables for verifiable Clifford circuits on *ibm_ithaca*. The ideal value can be obtained efficiently using Clifford circuit simulator and compared against the experiment results. As a first example, we set the transverse field in the Ising Hamiltonian to zero ($R_X(0) = I$) and evolve an initial state $|+\rangle^{\otimes 65} = (\mathsf{H}\,|0\rangle)^{\otimes 65}$, where H is the Hadamard gate. Inset of Fig. S3(a) illustrates the Hadamard gates applied at the beginning for the state preparation, and another Hadamard before measurement to perform a projective meausrement on $x-$axis. Since we apply $\mathsf{R_{ZZ}}(-\pi/2)$ rotational gate per Trotter step, and all the gates commute, every 4 Trotter steps makes a full $\mathsf{R_{ZZ}}(-2\pi)$ rotations and is equivalent to identity operation. Therefore, for any qubit q, the weight-1 observable $\langle X_q \rangle$ ideally recovers expectation value 1 every four steps. Note that the choice of initial state is different from the one in the main text, $|0\rangle^{\otimes 127}$. The particular choice of $|+\rangle^{\otimes 65}$ is to expose the circuit to phase errors, and similar experiment is performed and discussed in Sec. IV C for *ibm_kyiv*. Figure S3(a) shows the experimental results for averaged weight-1 observable, $\langle \bar{X} \rangle$. While the unmitigated observable shows a gradual decay from 1 with an increasing deviation for deeper circuits, the mitigated observables (blue symbol) show agreement with the ideal value even going up to 20 Trotter steps or CNOT depth 60.

Another Clifford angle of interest is $\theta_h = \pi/2$ ($R_X(\pi/2) = e^{-i\pi/4}\sqrt{X}$). We use Qiskit (`quantum_info.Clifford`) to efficiently find some stabilizers of the circuit, which have eigenvalues ± 1. The minimum weight of the stabilizers thus obtained grows for increasing Trotter steps, and here we select two such stabilizers for each Trotter step related by the 180° rotational symmetry of the connectivity of *ibm_ithaca* (Fig. S3(b) inset). Figure S3(b) shows absolute values of the estimated expectation values of the two minimum-weight stabilizers at each Trotter step. The unmitigated observable (empty symbol) clearly decays for increasing Trotter steps due to the accumulated errors arising from the increased circuit depth and weight of the observable.

III. NOISE MODEL INSTABILITY DUE TO TWO-LEVEL SYSTEMS (TLS)

Qubit-TLS interactions are a well known source of qubit coherence fluctuations. In this section, we first discuss data revealing the effect of TLS on error mitigated estimates of expectation values, and then present an additional experiment that repeatedly runs the noise-learning scheme to directly probe the drift of the device noise over time.

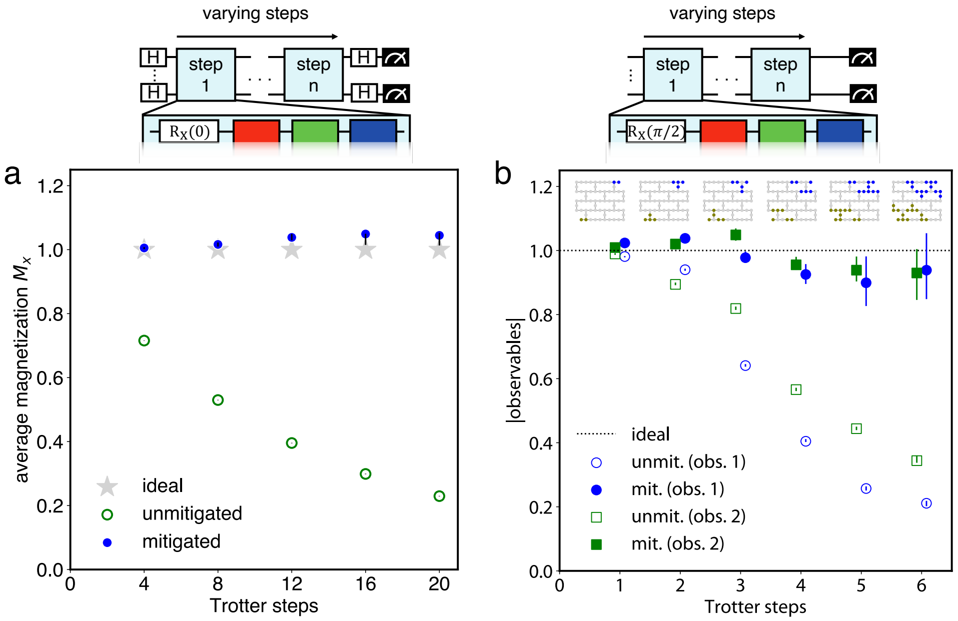

FIG. S3. **Experimental results on** ***ibm_ithaca*** **for Clifford circuits**. Similar experiments presented in the main text are carried out on *ibm_ithaca*. The magnetization M_x, computed by averaging weight-1 expectation values, is estimated for the circuit described in inset above (a). Here we have included the final H layer in the definition of the X measurements. (a) Results of verifiable Clifford circuits with $\theta_h = 0$. Mitigated estimates of M_x every 4 Trotter steps are in good agreement with the ideal value of 1 (stars). (b) Another verifiable Clifford circuit, with $\theta_h = \pi/2$. Here we measure selected stabilizers at each Trotter step whose true eigenvalue is 1. The error mitigation successfully estimates the ideal values up to Trotter step 6. Error bars indicate 68% confidence intervals obtained from 100 bootstrap configurations in all panels; some are smaller than the markers.

Both experiments were performed on *ibm_ithaca*.

A. TLS signatures in mitigated expectation values

Figure S4 illustrates the impact of the qubit-TLS interaction on error mitigated estimates of the same observable discussed in Fig. S2(a). Figure S4(a) focuses on $G = 1$ results, showing the fluctuation of $\langle X_2 \rangle$ in chronological order. The acquisitions were not evenly spaced in time, so the same data are plotted in Fig. S4(b) as a function of elapsed wall-clock time. Interleaved with these acquisitions were experiments to monitor qubit-TLS interactions by TLS spectroscopy [S11], plotted in Fig. S4(c). TLS spectroscopy monitors the excited state probability P_1 remaining after a fixed delay time of 20 μs as a quick estimator of T_1. A variable off-resonant Stark tone sweeps the qubit frequency, allowing us to obtain spectra of the qubit-TLS interactions. Dark regions in Fig. S4(c) indicate spectral dips in T_1 corresponding to strong qubit-TLS resonances. A clear dip develops for $\langle X_2 \rangle$ near elapsed time 99 to 113 hours in Fig. S4(b), where a corresponding feature is observed in Fig. S4(c). This correlation indicates that the large fluctuation of the unmitigated signal in Fig. S4(a) is likely due to the strong qubit-TLS interaction.

Figure S4(d) helps illustrate the impact of the qubit-TLS interaction on the mitigated results. The data processing performed in panel (d) differs from the standard procedure depicted in Sec. II. In this panel, each bin of 100 random circuit instances is extrapolated independently (blue circles) and then cumulatively averaged (blue line), rather than the standard procedure of bins being averaged and then extrapolated. Some additional scatter is expected in these results due to the limited number of circuit instances in each bin. However, the exponential extrapolation clearly fails to fully mitigate errors during the TLS dip, biasing the accumulated mean value away from the ideal value of 1.

In panel (e), we return to the standard procedure of averaging first and then extrapolating. If we include all of the data near the large dip, that data noticeably biases the extrapolated result. In contrast, panel (f) shows the result after rejecting outliers per Eq. 1, which improves the bias and reported uncertainty.

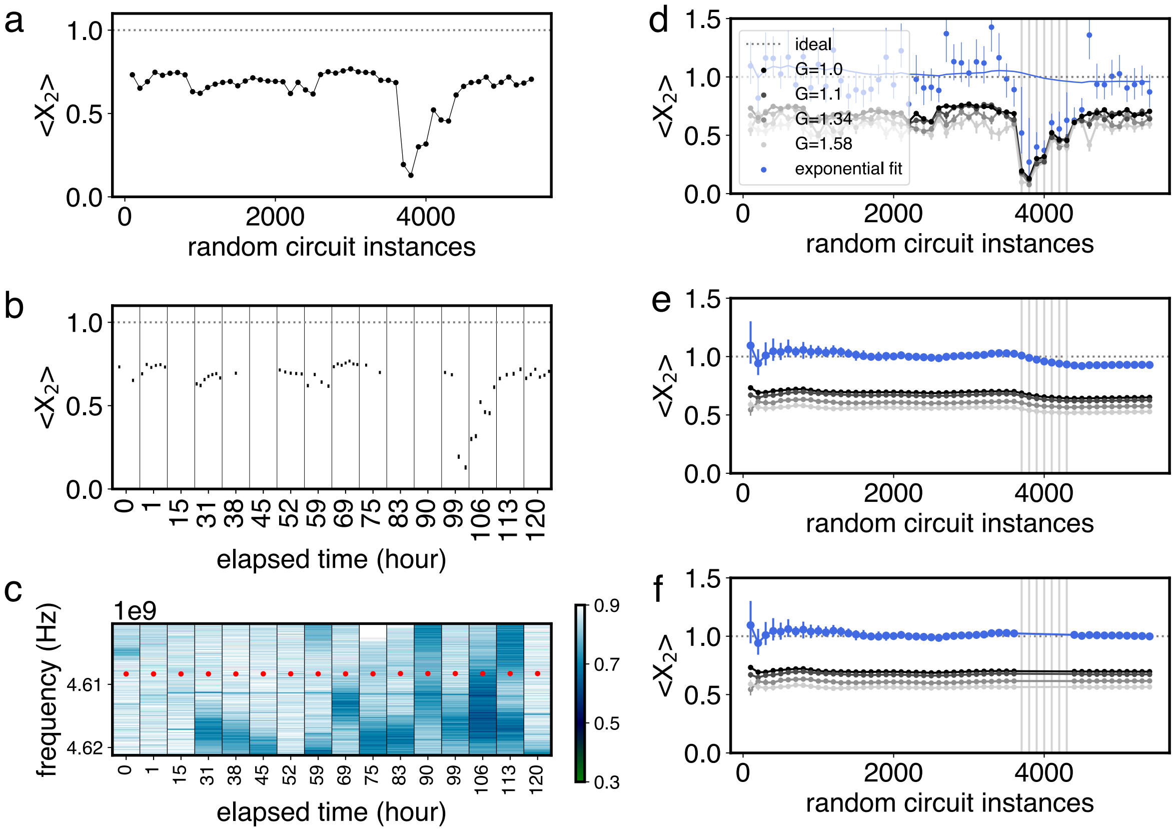

FIG. S4. **Impact of TLS on unmitigated and mitigated observable**. (a) A weight-1 unmitigated ($G = 1$) observable presented in Fig. S2(a). Each point represents 100 random realizations of Pauli/readout twirling. (b) The same data in (a) are now plotted against the start time of the mitigation task. Vertical lines indicate start times of the two-level system (TLS) spectroscopy. (c) TLS spectroscopy monitors excited state population P_1 after $\mathsf{R_X}(\pi)$ followed by $20\mu s$ delay. The decay is a proxy for T_1 and P_1 is collected near the original qubit frequency (red dots). Color bar indicates P_1 value and dark blue region indicates a strong qubit-TLS interaction. (d) Exponential extrapolation is performed for each point (blue circle). The accumulated average of each extrapolation results are presented (blue line). (e) The unmitigated observables are obtained from cumulative average, then the extrapolation is performed in exponential fit (blue line). (f) The unmitigated observables are obtained from cumulative average excluding the outliers indicated as vertical lines. We obtain extrapolated results with exponential fit and the results are shown in blue.

B. TLS signatures in learned noise models

The accuracy and stability of noise model play a crucial role in the efficacy of the error mitigation. With instabilities in coherence arising from qubit-TLS interaction as discussed above, a natural question is how these are captured in the noise learning. We monitored the stability of the noise model coefficients by repeatedly executing the noise learning procedure on *ibm_ithaca* for approximately 17 hours. The learning circuits contain a CNOT layer repeated 0, 2, 4, 8, 12, 24, or 48 times. We twirl the two qubit gate noise by inserting random Pauli gates before and after CNOT gates, along with random gates for readout twirling, producing 64 different random realizations. We then apply pre (post) rotational single qubit gates to collect all the relevant prepared (measured) Pauli bases to construct the sparse Pauli-Lindblad noise model [S8, S18]. We apply dynamical decoupling on qubits not participating in the layer of two qubit gates. Each learning experiment on all 65 qubits takes around 47 minutes on average.

Instead of visualizing all model coefficients, we define the single-qubit quantity $\gamma_i = \prod_{k\in\mathcal{K}_i} e^{2\lambda_k}$ where $\mathcal{K}_i$ is the set of model coefficient indices associated with weight-1 Pauli generators acting on the qubit i. Likewise, we also define local two-qubit quantity $\gamma_{i,j} = \prod_{k\in\mathcal{K}_{i,j}} e^{2\lambda_k}$ where $\mathcal{K}_{i,j}$ is the set of model coefficient indices associated with weight-2 Pauli generators acting on the qubit pair (i, j). Both γ_i and $\gamma_{i,j}$ are factors in the quantity γ that determines the sampling overhead of PEC [S18], which is discussed in the Methods section. Ideal gates on qubit i (qubit pair i, j) would produce $\gamma_i = 1$ ($\gamma_{i,j} = 1$), while noisy gates would produce values larger than 1. These quantities thus provide

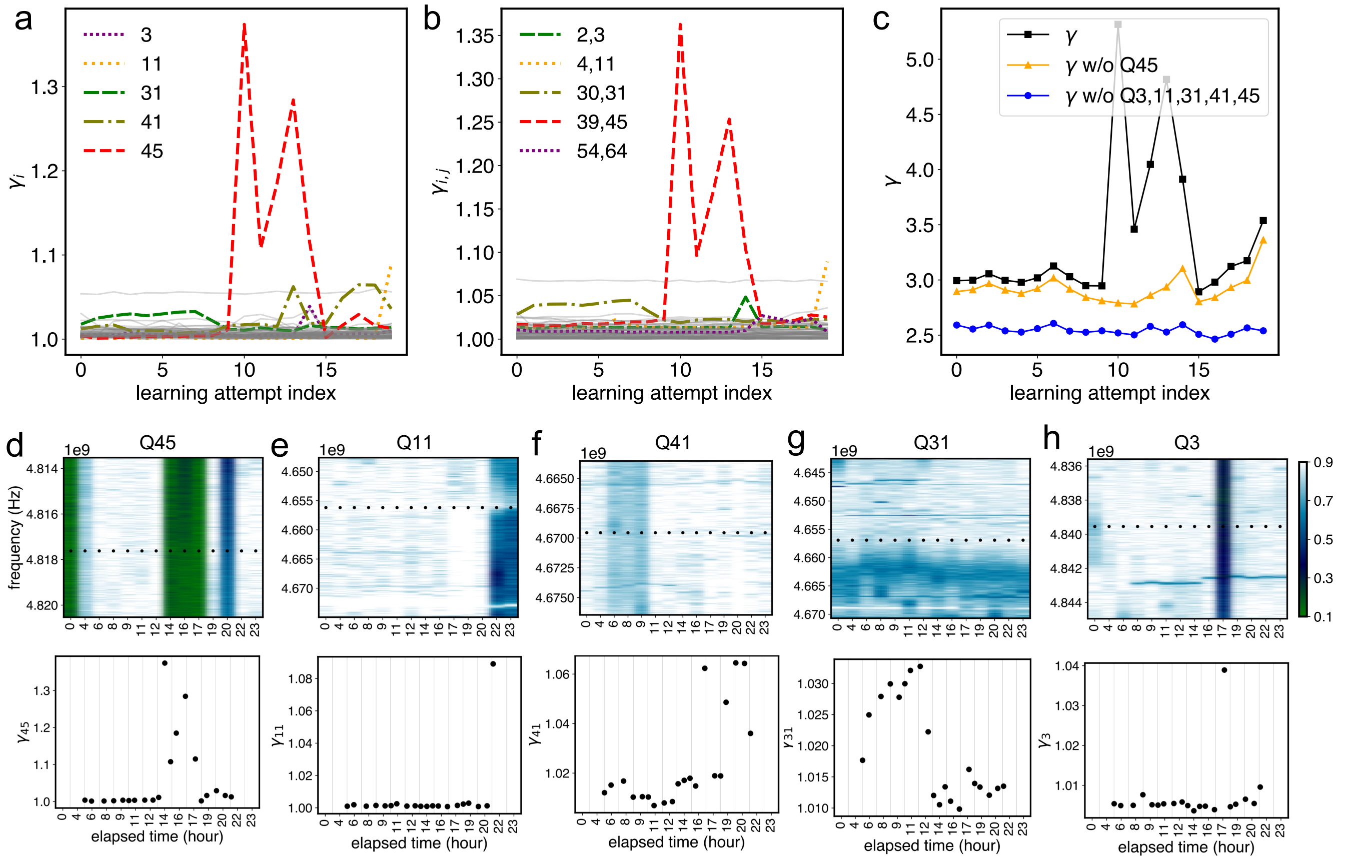

FIG. S5. **Experimentally measured stability on noise model.** The learning task is carried out on one particular set of two qubit gates for 21 consecutive times on *ibm_ithaca*. (a) A defined weight-1 noise parameter γ_i where $i \in \{0, \cdots, 64\}$ is qubit index. We measure its variability by the ratio of standard deviation and averaged value. The quantity that shows top five variability is highlighted. (b) A defined weight-2 noise parameter $\gamma_{i,j}$ where i, j are nearest neighboring qubit index. (c) Overall $\gamma = \Pi_i \gamma_i \Pi_{i,j} \gamma_{ij}$ parameter is plotted (rectangular symbol). γ shows reduced fluctuation once we exclude $i, j = 45$, and becomes relatively stable if we exclude $i, j = 3, 11, 31, 41, 45$. (d-e) We plot TLS spectroscopy that is monitored during the experimental period on top pannel. The bottom panel shows the corresponding γ_i, which is binned based on the experiment start time. The color bar is P_1 value measured using the same protocol described in Fig. S4.

convenient ways of monitoring noise model fluctuations local to a specific qubit or pair of qubits.

Figure S5(a, b) shows examples of fluctuations in γ_i and $\gamma_{i,j}$, respectively. In each panel, we show the five qubits (qubit pairs) with the largest relative fluctuations, computed as the standard deviation divided by the mean value. We find that most of the qubit indices identified in Fig. S5(a) also appear in Fig. S5(b), suggesting the overall error is dominated by noise localized to a few particular qubits. Figure S5(c) shows the quantity $\gamma = \prod_{k \in \mathcal{K}} e^{2\lambda_k}$, related to the sampling cost of the layer, where $\mathcal{K}$ spans all model parameters (squares). Excluding the most prominent fluctuator, qubit 45, significantly reduces the fluctuation of the overall γ (triangles). Removing all model errors associated with the top five fluctuators in Fig. S5(a), i.e. excluding all $i, j \in \{3, 11, 31, 41, 45\}$, further stabilizes and reduces γ.

Figure S5(d-h) shows TLS spectroscopy near the frequency of the specified qubit (upper panel) and the corresponding γ_i (lower panel) over time. The bottom panel γ_i is binned based on the TLS spectroscopy time and the start time of the learning task. Dark vertical bands in panels (d,e,h) indicate broad suppression of P_1 due to strong qubit-TLS interactions, correlated with elevated γ_i in the bottom panel. Panel (f) also shows correlation of broader P_1 suppression with the moderate elevation in γ_{41}. We observe more significant elevation of γ_{41} when a spectrally narrow TLS feature develops close to Q41 frequency. Similar behavior is observed in (g), where a TLS (dark horizontal band) drifts into resonance with the qubit around 4-9 hours in elapsed time. These observations help clarify how TLS spectral dynamics are a major contributor to instability of the noise models.

It is challenging to prevent qubit-TLS interaction as the physical origin of TLS is not fully understood and superconducting transmon qubits are generally exposed to multitudes of TLS [S19, S20]. Nonetheless, Fig. S5 highlights that addressing strong qubit-TLS interactions in superconducting qubits will be crucial for the stability of learned

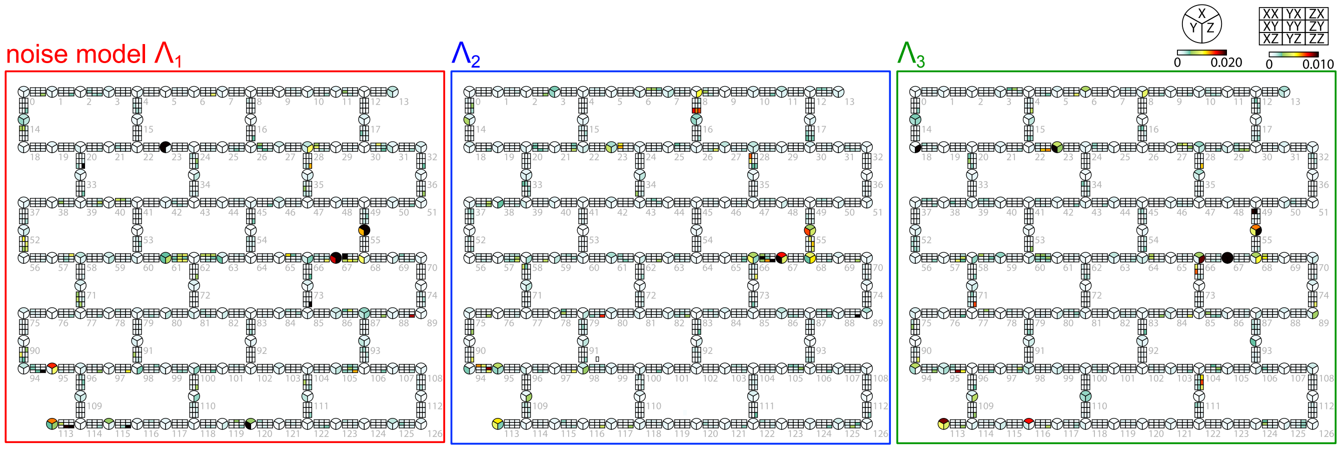

FIG. S6. **Example noise models for the three CNOT layers.** The three noise models shown partly obscured in Fig. 1c are shown here in full.

noise model, and therefore for extending the reliability of error mitigation performance on large scale processors.

IV. ADDITIONAL DETAILS ON THE TROTTER EXPERIMENTS ON *ibm_kyiv*

This section provides further supporting details regarding the Trotter-simulation experiments in the main text, which were run on *ibm_kyiv*.

A. Noise learning and random-circuit generation

The noise model of *ibm_kyiv* is obtained following a protocol similar to that outlined in Sec. III B. The learning circuits consists of a CNOT layer repeated 0, 2, 4, 8, 12, or 48 times, and 40 random circuit realizations for Pauli/readout twirling. The averaged wall-clock time for the learning task takes around 34 minutes. We sequentially learn the three layers of two qubit gates described in Fig. 1 in the main text. The three example noise-models shown overlapping in Fig. 1(c) are displayed in full in Fig. S6. We refreshed the noise model by running the learning procedure again on average every ~ 7 hours 29 minutes. For example, learning task was performed twice for Fig. 2(c) and four times for Fig. 3(a,b).

We carry out the error-mitigation experiment in Fig. 2(c) by compiling the circuits for $G = 1, 1.2, 1.6$ and $0, 4, 8, 12, 16, 20$ Trotter steps, producing for each configuration 100 random circuit realizations on Pauli/readout twirling and insertion of Pauli gates for error amplification. The compiled circuits are submitted to the quantum backend in a random order so that all the relevant circuits are exposed to the similar noise environment during the experiment. The experiment is repeated 20 times with different random seeds in order to accumulate 2000 circuit instances.

Experiments in Fig. 3(a-b) are performed by compiling the circuits for $G = 1, 1.2, 1.6$, and $0, 5$ Trotter steps at a particular choice of θ_h. We use two distinct measurement bases (one for weight-1 observable, the other for weight-10 observable) for 100 random circuit realizations on Pauli/readout twirling and insertion of Pauli gates for error amplification. The batched tasks are submitted for various θ_h first then the experiments are repeated 20 times with different random circuit realizations to accumulate 2000 circuit instances in total. This way of batching allows us to expose experimental results for various θ_h to relatively similar temporal variation of device characters.

Experiments in Fig. 3(c) and Fig. 4(a-b) are performed in a similar fashion with Fig. 3(a-b), but using one distinct measurement basis for the observable of interest. he tasks are similarly batched per 100 random circuit realizations and submitted for various θ_h. Experiments in Fig. 3(c) and Fig. 4(b) are performed with $G = 1, 1.2, 1.6$, while $G = 1, 1.3, 1.6$ is used for Fig. 4(a). We could not find any discernible difference in extrapolation quality for two different choices of gain factor. We accumulated more random circuit instances for certain angles, for instance near $\theta_h = \pi/2$ in Fig. 4(a), in order to have better confidence interval.

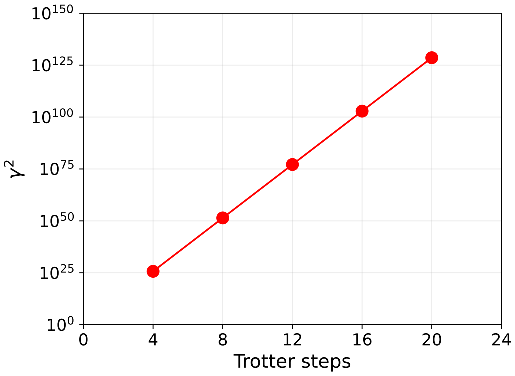

FIG. S7. **Estimated sampling costs using a naive PEC implementation to mitigate the Trotter circuits of the main text.** These astronomical values suggest that such a mitigation scheme would be untenable here, motivating the use of methods expected to have lower overhead such as ZNE or lightcone-optimized PEC.

B. Predicted sampling costs of a naive PEC implementation

In this section, we estimate what the sampling cost would be if we used a naive PEC implementation (i.e. not optimized by lightcone analysis [S17]) instead of ZNE to mitigate errors in Trotter experiments of the main text. Six different noise models, each including all three layers, were learned and employed to obtain data for Figs. 2 and 3. Defining γ_l as the γ (see Methods section for more details) of a circuit containing one copy of the CNOT layer l, the median (15%, 85%) γ_l from 29 separate learning attempts for the three layers are 10.60 $(-1.37, +3.3)$, 12.00 $(-1.24, +6.14)$, and 12.88 $(-3.83, +2.89)$. Applying PEC to a circuit with 20 Trotter steps (CNOT depth 60) would thus incur a sampling cost of $\gamma^2 = (\gamma_1\gamma_2\gamma_3)^{40} \sim 10^{128}$. Values are plotted as a function of circuit depth in Figure S7. We see that ZNE performed well in the main-text experiments despite the presence of hardware noise predicted to make a naive PEC implementation unaffordable, highlighting the importance of choosing an error mitigation scheme suitable for a given computational task and available quantum hardware.

C. Magnetization measurements with $\theta_h = 0$ circuits

Here we further detail the Clifford circuit experiments in Fig. 2(c) to provide better understanding on device-wide error mitigation results for varying Trotter steps. In addition, we show the effectiveness of the exponential extrapolation compared to first order polynomial extrapolation. While exponential extrapolation provides minimal bias on average, first order extrapolation results clearly show increasing deviation from the ideal value for deeper and more noise-prone circuits. The last point is discussed further in Sec. V.

Figure S8(a) presents the same data from Fig. 2(c) up to 12 Trotter steps. Panels (b-d) detail the data underlying the points in (a), including the unmitigated values ($G = 1$) along with the results of first-order polynomial and exponential extrapolations, and finally the *best* extrapolation as defined in Sec. II. During the experiment, we identified strong qubit-TLS interactions affecting qubit 23, 55, and 67 that manifest as low-flying outliers in the unmitigated data. As expected, uncertainties generally increase with circuit depth (Fig. S8(b-d)), as more errors accumulate during the longer circuits; in a few cases this leads to unphyiscally large observable values for exponential extrapolation with large fit uncertainty. Note that the resultant mean value of the exponential extrapolation in Fig. S8(d) is biased by a data point with poor fit and this results in an unphysically large observable estimate, outside of the plotting range. In this case, *best* fit selects first order polynomial extrapolation such as the outliers Q67 in Fig. S8(c) and Q23,67 in Fig. S8(d) for experimental value. A similar situation happens for bootstrapped values; portion of the bootstrapped instances select exponential fit while another portion of the instances choose first order extrapolation, resulting in large error bar. We leave optimization of how to threshold extrapolation method selection to best trade off bias and variance for future work.

For completeness, Fig. S3(e-h) show results of an analogous experiment with an additional layer of Hadamard gates at the start and end of each circuit (inset), similar to Fig. S3(a). Here the unmitigated results exhibit greater bias and variance compared to those in Fig. S3(a), which we attribute to the Hadamard gates making the state sensitive to phase errors. Nonetheless, exponential ZNE still recovers comparable accuracy. In contrast, the biases of polynomial fits grow significantly with circuit depth (more pronounced for the circuit with Hadamards), supporting the preference

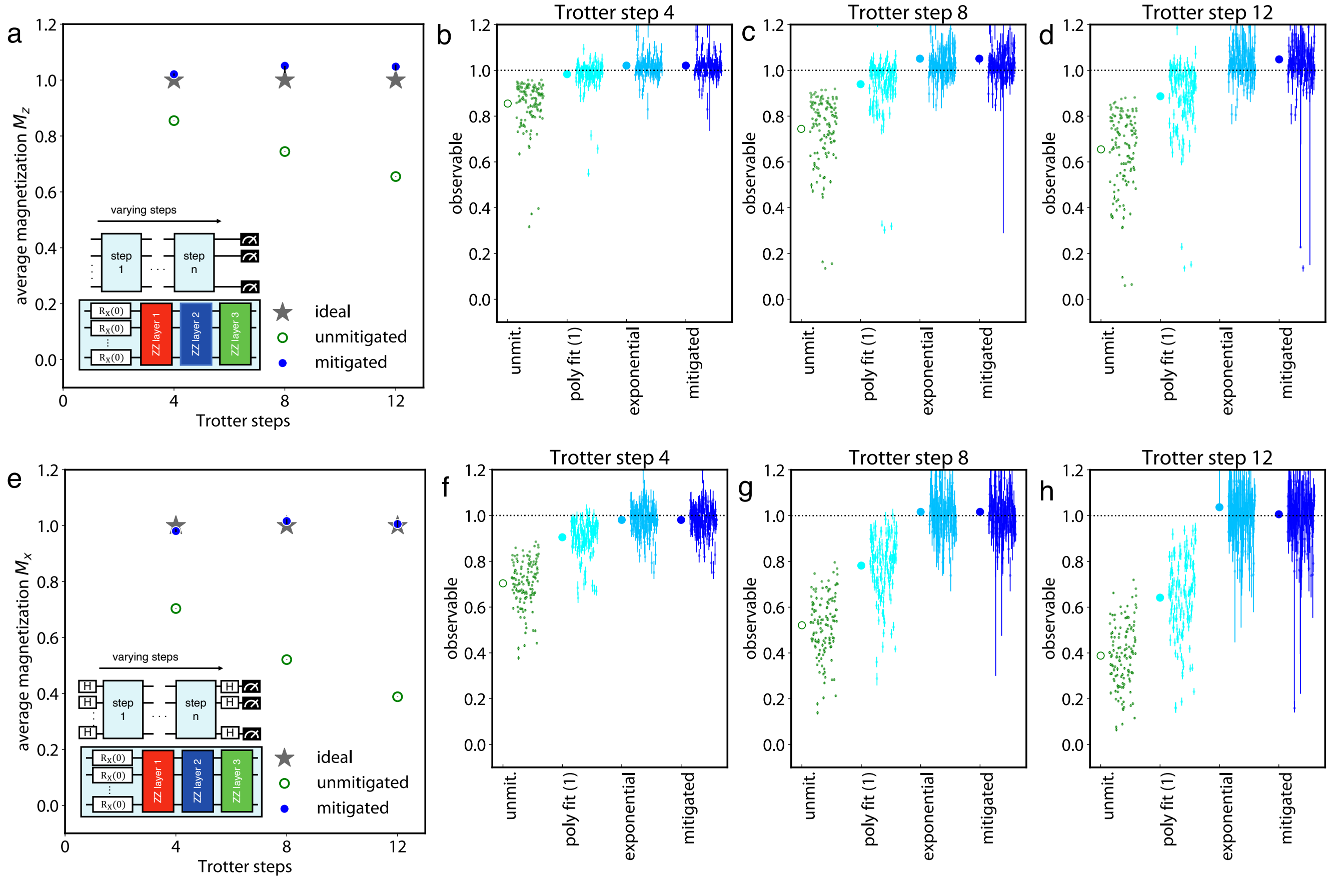

FIG. S8. **Further details of mitigated magnetizations with $\theta_h = 0$ on *ibm_kyiv*.** (a) Global magnetization M_z as a function of Trotter steps of the inset circuit. The data are reproduced from Fig. 2(c). (b-d) Distributions for unmitigated, polynomial extrapolation, exponential extrapolation, and the *best* fit results after 4, 8, and 12 Trotter steps in panels (b-d) respectively. (e-h) Similar to the top row, but with initial and final Hadamard gates that increase susceptibility to phase errors. Error bars indicate 68% confidence interval obtained from 100 bootstrap configurations in all panels; some are smaller than the markers.

for exponential extrapolation in most cases, as further motivated in Sec. V.

V. EXPONENTIAL EXTRAPOLATION

In this section we study how the observable expectation values change with different scaling factors of noise amplification and suppression. For Clifford circuits we show that the expectation values can be described by exponential curves. For circuits with non-Clifford gates, this generally changes to sums of exponentials. A different derivation in [S21] resulted in the same multi-exponential form.

A. Noise scaling

As a preliminary, let $\mathcal{U} = U \cdot U^\dagger$ be a linear operator and denote by $\mathbb{P}_n = \{P_i\}_{i=1}^{4^n} = \{I, \sigma_x, \sigma_y, \sigma_z\}^{\otimes n}$ the set of n-qubit Pauli operators. The Pauli transfer matrix (PTM) corresponding to $\mathcal{U}$ indicates, informally, how much of Pauli P_j it transforms to Pauli P_i. The matrix $M_\mathcal{U}$ therefore has elements

$$M_\mathcal{U}[i,j] = \frac{1}{2^n}\,\mathrm{tr}[P_i \mathcal{U}(P_j)], \quad \text{with} \quad i,j \in [1, 4^n].$$

A useful property of Pauli transfer matrices that we will use below, is that the PTM of a product of linear operators $\mathcal{A}$ and $\mathcal{B}$ satisfies $M_{\mathcal{AB}} = M_{\mathcal{A}} M_{\mathcal{B}}$. For Clifford operators $\mathcal{G}$, $M_{\mathcal{G}}$ is a signed permutation matrix. That is, each row and column of $M_{\mathcal{G}}$ contains exactly one element with value ± 1. In particular, we define the non-zero element in column j to be $\sigma_{\mathcal{G}(P_j)} \in \{-1, +1\}$, such that $T_{\mathcal{G}}(P) := \sigma_G(P)\mathcal{G}(P) \in \mathbb{P}_n$ for any $P \in \mathbb{P}_n$. That is, application of $\mathcal{G}$ on P results in Pauli $T_{\mathcal{G}}(P)$ with sign $\sigma_{\mathcal{G}}(P)$. We can express the noisy implementation of an operator $\mathcal{U}$ as $\tilde{\mathcal{U}} = \mathcal{U} \circ \Lambda_{\mathcal{U}}$, where $\Lambda_{\mathcal{U}}$ represents a noise channel. By virtue of Pauli twirling, we can assume, without loss of generality, that $\Lambda_{\mathcal{U}}$ is a Pauli channel:

$$\Lambda_{\mathcal{U}}(\rho) = \sum_{j=1}^{4^n} c_{\mathcal{U}}[j] P_j \rho P_j^{\dagger}, \tag{3}$$

with $c_{\mathcal{U}}[j] \geq 0$ and $\|c_{\mathcal{U}}\|_1 = 1$. The Pauli transfer matrix $M_{\Lambda_{\mathcal{U}}}$ is diagonal with elements

$$f_{\Lambda_{\mathcal{U}}}(P_i) = \frac{1}{2^n} \operatorname{tr}[P_i \Lambda_{\mathcal{U}}(P_i)] = \sum_{j=1}^{4^n} \mu(P_i, P_j) c_{\mathcal{U}}[j],$$

where $\mu(P_i, P_j)$ is 1 if P_i and P_j commute (that is, $P_i P_j = P_j P_i$), and -1 otherwise. A special class of structured Pauli channels can be represented by the Pauli-Lindblad error model. Given such a model, Λ, with model parameters $\{\lambda_k\}$ and associated Pauli terms $\{S_k\}$, we have fidelities [S18]

$$f_{\Lambda}(P_i) = \exp\Big(\sum_k \lambda_k(\mu(S_k, P_i) - 1)\Big) > 0.$$

Scaling the model coefficients λ_k by a factor β results in the noise operator Λ^{β} with (diagonal) Pauli transfer matrix $F_{\Lambda^{\beta}} = F_{\Lambda}^{\beta}$. Although the resulting operator is no longer a Pauli channel for $\beta < 1$, it can still be applied, on average, using quasi-probabilistic sampling. For general Pauli channels, we can form $\Lambda_{\mathcal{U}}^{\beta}$ by exponentiating the fidelities and apply the Walsh-Hadamard transformation to obtain updated channel coefficients $c_{\mathcal{U}}$.

B. Noisy Clifford circuits

The ability to apply Λ^{β} allows us to control the noise for a gate. For instance, premultiplying a noisy Clifford gate $\tilde{\mathcal{G}} = \mathcal{G} \circ \Lambda$ by Λ^{β}, with $\beta = \alpha - 1$, results in an operator $\tilde{\mathcal{G}}_{\alpha}$ with transfer matrix $M_{\mathcal{G}} F_{\Lambda} F_{\Lambda}^{\alpha-1} = M_{\mathcal{G}} F_{\Lambda}^{\alpha}$. In probabilistic error cancellation (PEC), this property is leveraged to cancel gate noise by choosing $\alpha = 0$, since $F_{\Lambda}^{0} = I$. In probabilistic error amplification scenario, we choose $\alpha \in (0, 1)$ or $\alpha > 1$ to reduce, respectively amplify, the gate noise. Now, consider a quantum circuit that is implemented using a series of noisy Clifford operators $\tilde{\mathcal{G}}_{\ell} = \mathcal{G}_{\ell} \circ \Lambda_{\ell}$ with $\ell = 1, \ldots, L$. The overall Pauli transfer matrix of the circuit is given by a product of diagonal fidelity matrices and (signed) permutation matrices. As a consequence, Pauli terms never mix, and we can therefore follow the transformation of an arbitrary Pauli operator P_0. Given an initial state ρ_0, the initial weight of Pauli P_0 is given by $w_0 = \operatorname{tr}[P_0 \rho_0]$. For instance, for $\rho_0 = |0\rangle\langle 0| = \frac{1}{2}(I + \sigma_z)$ and $P_0 = \sigma_z$ we find $\alpha_Z = 1$ with initial weight w_0. We then successively apply the gates. At step ℓ we apply gate $\tilde{\mathcal{G}}_{\ell}$, which changes the Pauli to $P_{\ell} = T_{\mathcal{G}_{\ell}}(P_{\ell-1})$ and updates the weight to $w_{\ell} = \sigma_{\mathcal{G}_{\ell}}(P_{\ell-1}) f_{\Lambda_{\ell}}^{\alpha}(P_{\ell-1})$. At the end of the circuit we often want to determine the expectation of some Pauli observable O. Given the unique P_0 such that $P_L = O$, the expectation value is given by

$$\langle O \rangle_{\rho_0,\alpha} = \frac{1}{2^n} \operatorname{tr}\left[O\left(\prod_{\ell=1}^{L} \tilde{\mathcal{G}}_{\ell}\right)(\rho_0)\right] = \alpha_0 \prod_{\ell=1}^{L} \sigma_{\mathcal{G}}(P_{\ell-1}) f_{\Lambda_{\ell}}^{\alpha}(P_{\ell-1}) = \left(\alpha_0 \prod_{\ell=1}^{L} \sigma_{\mathcal{G}}(P_{\ell-1})\right) \cdot \left(\prod_{\ell=1}^{L} f_{\Lambda_{\ell}}(P_{\ell-1})\right)^{\alpha}.$$

The observable expectation value $\langle O \rangle_{\rho_0,\alpha}$ is therefore an exponential function of the form $v \cdot s^{\alpha}$, where v is equal to the noise-free observable expectation value $\alpha_0 \prod_{\ell=1}^{L} \sigma_{\mathcal{G}}(P_{\ell-1}) = \frac{1}{2^n} \operatorname{tr}\left[O\big(\prod_{\ell=1}^{L} \mathcal{G}_{\ell}\big)(\rho_0)\right]$. As a result, extrapolation based on an exponential fit through the observable values $\langle O \rangle_{\rho_0,\alpha}$ at several α values yields a perfectly error-mitigated observable expectation value (simply evaluate the fitted exponential at $\alpha = 0$). For simplicity, we have omitted in the above discussion, the effect of readout errors. With appropriate twirling of the readout [S8], these error appear as multiplication of v by a readout fidelity. When known, this factor can be eliminated by scalar division.

C. Non-Clifford gates

Quantum circuits that start in a basis state and contain only Clifford operators can be simulated efficiently classically [S22], and practically meaningful quantum circuits must therefore contain non-Clifford gates. We now show that expectation values $\langle O\rangle_{\rho_0,\alpha}$ for general circuits are sums of exponentials. The fundamental reason for this is due to the fact that Pauli transfer matrices of non-Clifford operators no longer have a permutation structure. For instance, the PTM for the single-qubit operator for unitary z-rotation $R_z(\theta) = e^{-i\frac{\theta}{2}\sigma_z}$ is given by

$$M_{\mathcal{R}_z(\theta)} = \begin{bmatrix} 1 & 0 & 0 & 0 \\ 0 & \cos(\theta) & -\sin(\theta) & 0 \\ 0 & \sin(\theta) & \cos(\theta) & 0 \\ 0 & 0 & 0 & 1 \end{bmatrix}.$$

This means that application of the operator on σ_x gives $\mathcal{R}_z(\theta)(\sigma_x) = \cos(\theta)\sigma_x + \sin(\theta)\sigma_y$, which is no longer a single Pauli. Now, suppose we have a state ρ obtained by applying a series of noisy Clifford gates with noise-scaling factor α. Given Pauli terms $P_x = \sigma_x \otimes P$ and $P_y = \sigma_y \otimes P$ with weights $w_x = \mathrm{tr}[P_x\rho]/2^n$ and $w_y = \mathrm{tr}[P_y\rho]/2^n$, it follows from the discussion in the previous section that w_x is of the form $v_x s_x^\alpha$, and likewise that $w_y = v_y s_y^\alpha$. Assuming for simplicity that $\mathcal{R}_z(\theta)$ can be applied to the first qubit in a noiseless manner (or that the noise is already included in ρ), we find that

$$\tfrac{1}{2^n}\,\mathrm{tr}[(\sigma_x \otimes P)\cdot \mathcal{R}_z(\theta)(\rho)] = \cos(\theta)w_x - \sin(\theta)w_y = \cos(\theta)v_x s_x^\alpha - \sin(\theta)v_y s_y^\alpha.$$

This updated weight is sum of exponentials and, except in the special case where $s_x = s_y$, cannot be written as a single exponential. Subsequent Clifford operators premultiply this sum by other exponential functions, resulting in a new sum of exponentials that retains the same number of terms. Application of other non-Clifford operator may fortuitously combine some terms, but generally results in additional terms in the sum of exponentials. When the base terms s in this sum are similar, the curve can be well approximated using a single exponential. The unknown number of exponential terms makes the non-Clifford circuits fundamentally more challenging to mitigate with ZNE, adding to the importance of experimental tests in that regime.

VI. CLASSICAL SIMULATION ALGORITHMS

We have discussed classical simulation methods in Methods section of the main text; brute-force simulation and tensor network methods in order of increasing complexity. Here, we discuss further details on matrix product states (MPS) and isometric tensor networks (isoTNS) methods.

A. Tensor network methods

1. Matrix Product States

When representing a two-dimensional (2D) quantum state by a 1D matrix product state (MPS), one can take advantage of a favorable $\mathcal{O}(\chi^3)$ scaling of complexity with bond dimension χ and three decades of algorithm development [S23–S26]. However, an exponential scaling of computational complexity with the smallest linear dimension of the 2D layout is unavoidable. This manifests in different ways depending on how the circuit evolution is performed. Additionally, initially local nearest-neighbor interactions in 2D become long-range in 1D. Below we discuss several different approaches to simulating quantum dynamics of 2D systems with MPS.

Before discussing two different approaches for applying multi-qubit gates to an MPS, note that the single-qubit unitaries can be applied directly to the MPS without increasing the bond dimension. This is because single qubit unitaries cannot increase entanglement. The application of the single-qubit unitary does not destroy the canonical form of the MPS.

To simulate a 2D quantum state with an MPS, one must first choose how to wind the MPS through the 2D Heavy-Hexagon lattice. Local connections in 2D become non-local in the 1D ordering, and the choice of ordering affects entanglement of the resulting quantum state, as we generally expect nearest-neighbor qubits in 2D to be strongly entangled. Two possible options are shown in Figure S9. The 'Ring' order in (a) is typically used in simulations of 2D systems on cylinders; the MPS is chosen to wind along the y axis, as the dimension L_y in the y-direction is chosen to be less than L_x, and thus entanglement between rows is generically more than entanglement between columns.

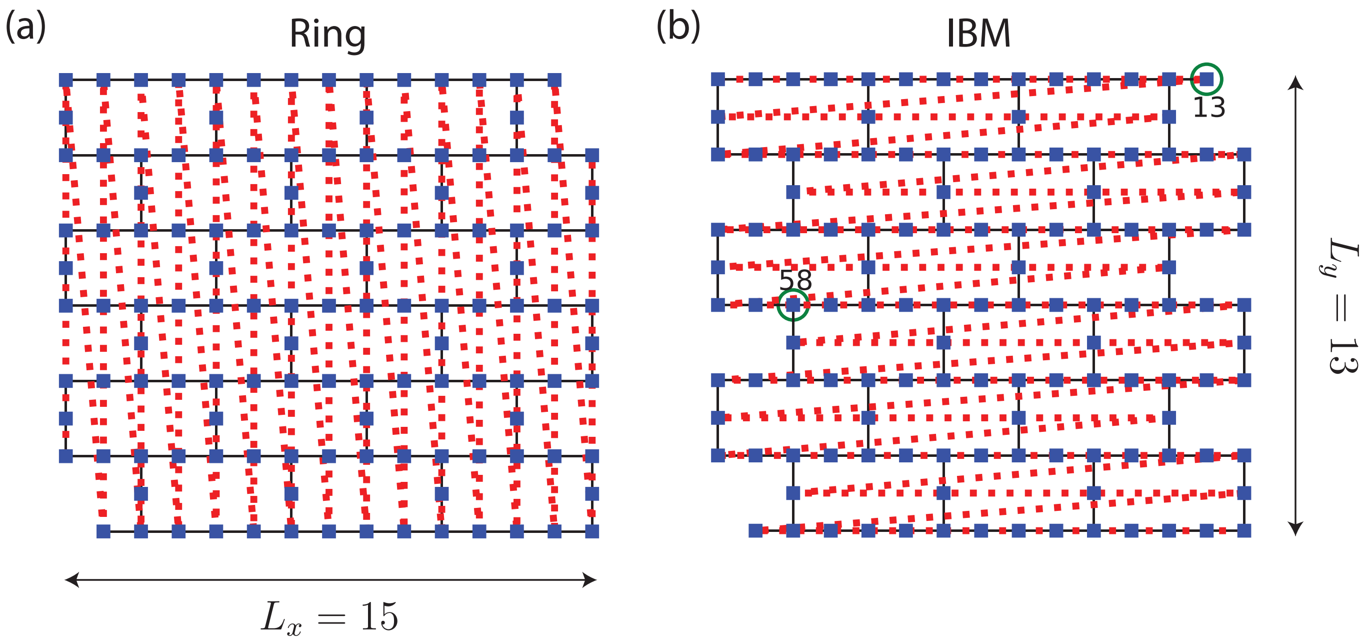

FIG. S9. Two possible ordering of the 1D MPS through the 2D Heavy-Hex lattice, deformed to fit in a 15×13 2D square lattice with $13 * 15 - 127 = 68$ missing sites. (a) 'Ring' ordering typically used in studies of two-diemsional models on a cylinder. This ordering can be used for gate-based TEBD approaches. (b) 'IBM' ordering used to construct MPO representing layer of two-qubit gates. Circled sites 13 and 58 are the starting points for the weight-10 and weight-17 operators considered in the main text.

However, as the heavy hexagon lattice can be viewed as a square lattice with missing connections, another natural choice is the 'IBM' ordering in (b). This ordering of the MPS reflects that the majority of nearest-neighbor couplings are within the rows. This is the ordering (arbitrarily) used to label the qubits in the 127-qubit device. As we will discuss below, the optimal choice of ordering is determined by the method used to evolve the quantum state with a layer of two-qubit gates.

The standard approach to simulating local dynamics on tensor networks is the Time Evolving Block Decimation (TEBD) algorithm, which naturally encompasses gate-based evolution [S24, S27]. Local unitaries are applied to the MPS, increasing the bond dimension, and then the bond dimension is truncated back to χ, incurring an error. Note that not all Heavy-Hexagon nearest-neighbor interactions are local in the 1D MPS (e.g. interactions within rows (columns) in the 'Ring' ('IBM') order). While `SWAP` gates can be used to reshuffle qubits in the chain, each swap gate increases the bond dimension and thus incurs an error. An alternate approach is to simulate a linear chain of qudits representing a set of qubits of the original layout. This approach was recently used to simulate random circuit sampling of 54 qubits with modest computational cost [S28, S29]. For the 'Ring' ('IBM') order in Figure S9(a (b)), columns (rows) would be collapsed to form a linear chain of 15 (13) qudits. The qudits would have max dimension $d = 2^{10} = 1024$ ($2^{15} = 32768$). Now, no swap gates are needed. Interactions within the column (row) can be applied without error, while interactions between columns (rows) are now 2-local. However, the computational cost of TEBD is $\mathcal{O}(\chi^3 d^3)$, and the 2-local gates are $d^2 \times d^2$ matrices. Thus for the 127-qubit device considered, this method requires far too much memory, regardless of MPS ordering.

A second approach that does not require grouping qubits into qudits is to directly simulate time evolution with long-range interactions. Operators with long-range interactions can be efficiently encoded as matrix product operators (MPO), which are commonly used to represent Hamiltonians in the celebrated ground state Density Matrix Renormalization Group (DMRG) algorithm. Several algorithms exist for performing time evolution given an MPO representation of a Hamiltonian [S24]. As the two-qubit R_{ZZ} gates are the matrix exponentiation of a Hamiltonian $H = J\sum_{\langle i,j\rangle} Z_i Z_j$, with the cofficient J determined by the angle θ_h, we could start with an MPO representation of a sum of nearest-neighbor (in 2D) ZZ gates. However, since we explicitly know the time evolution unitary to apply, namely $\prod_{\langle i,j\rangle} \mathsf{R}_{Z_i Z_j}(-\pi/2t)$, we instead work directly with MPOs representing this unitary. Note that the bond dimension of this unitary is exponential in linear system dimension; for the 'IBM' ordered MPS, the needed bond dimension is 64, while it is 256 for the 'Ring' ordering. Thus we use the 'IBM' ordering for all MPS simulations.

As in the experiment, for each Trotter step, we represent each layer of two-qubit R_{ZZ} gates acting on all nearest-neighbor pairs as 3 layers of parallelizable gates. Each layer is represented by a separate MPO, M_i. The action of the

entire layer of gates on state $|\Psi\rangle$ is simply the product of the MPOs acting on the state, $M_3 M_2 M_1 |\Psi\rangle$. As all the gates commute, the ordering of the laters and the partitioning of the gates into three disjoint sets is not physically relevant. We do not choose the same 3 disjoint sets as used in the experiment, which are shown pictorially as different colored bonds in Figure 1(b). We choose to use three MPOs instead of a single MPO representing the entire layer of two-qubit gates due to ease of construction. To apply an MPO to an MPS, we use a subroutine in the tensor network literature called MPO-MPS contraction. We adopt a variational compression for MPO-MPS contraction as this is less memory intensive than more straightforward SVD compression [S25].

2. *2D Isometric Tensor Networks*

Given inherent limitations of MPS methods for simulating 2D circuits, a true 2D method is required to scale to large system sizes. isoTNS are a restriction of two-dimensional projected entanglement pair state (PEPS) tensor networks [S23, S30–S34] such that each tensor in the network is an isometry [S35]. Diagramatic representation of an isoTNS and isometric conditions are shown in Figure S10.

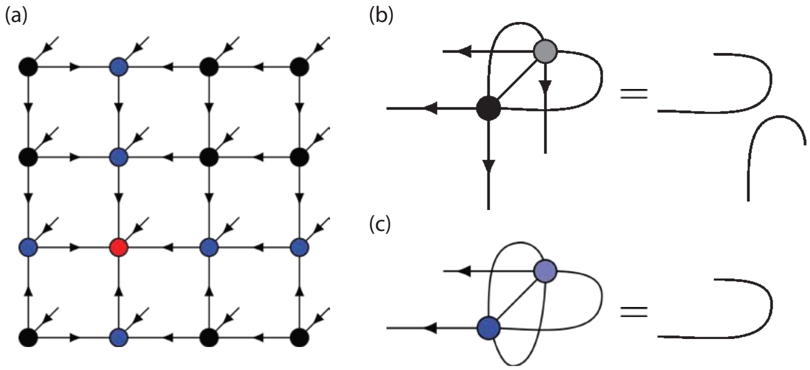

FIG. S10. Graphical representation of a isometric tensor network for 16 spins on a 4×4 square lattice. The leg sticking out the page is the physical basis state $\sigma_{(i,j)}$ on each site (i, j). The contraction of the connected legs for the 16 tensors gives the rank-16 tensor of wave function coefficients. The arrows on the legs denote the isometry conditions. For each tensor and its conjugate, contracing over the legs with incoming arrows yields an identity on the legs with outgoing arrows. This is shown in (b) for a black tensor with 2 outgoing legs and in (c) for a tensor with 1 outgoing leg. The red tensor in the isoTNS is the *orthogonality center* (OC).

The additional isometric structure makes both time evolution via TEBD and the evaluation of single-site observables more efficient ($\mathcal{O}(\chi^7)$) than is possible with standard PEPS methods ($\mathcal{O}(\chi^{12})$ and $\mathcal{O}(\chi^{10})$). This reduction in computational cost comes at the expense of additional approximation error from imposing the isometric constraints; yet ground state optimizations are competitive with density matrix renormalization group (DMRG) and quantum Monte Carlo (QMC) [S36, S37], and a large class of topologically ordered states have been shown to have an exact isoTNS representation with finite bond dimension in the thermodynamic limit [S38].

Isometric tensor networks were introduced in [S35] and discussed in more detail in [S36]. Here we will not introduce the method but instead discuss changes in network structure due to the Heavy-Hexagon lattice and expectation value techniques for the weight-10 and weight-17 operators.

Algorithms for isometric tensor networks have been developed for 2D square lattices. So we embed the Heavy-Hex lattice inside a 2D square lattice and add "fake" sites that are not coupled to the physical qubits by circuit unitaries. The 127 qubit geometry is embedded in a 15×13 square lattice with 68 fake sites. We initialize these fake sites in the $|0\rangle$ state. We check that $\langle Z\rangle = 1$ and $\langle X\rangle = \langle Y\rangle = 0$ on these sites after circuit layer to ensure that the fake qubits are not being coupled to the physical qubits.

For a generic PEPS, calculating expectation values of single-site observables requires costly, approximate calculations involving the entire network; the cost of measurements scales as $\mathcal{O}(\chi^{10})$ with bond dimension χ. For isoTNS, we can take advantage of the isometric structure to greatly reduce the cost of single-site expectation values to $\mathcal{O}(\chi^7)$. Consider measuring an operator $\mathcal{O}$ on site $(2, 2)$ of a 4×4 network, as shown in Figure S11. Site $(2, 2)$ is the *orthogonality center* (OC), denoted graphically by the red tensor. The OC has only incoming isometry arrows and is analogous to the OC in 1D MPS. Calculating the expectation value of a single-site observable on the OC becomes a local procedure only involving the tensor on site $(2, 2)$. This approach can be applied to any single-site observable on any-site, given the ability to reconfigure the isoTNS and move the OC to any site in the network. The movement of the OC is achieved

by the *Moses Move* algorithm introduced in [S35] which has a computational cost of $\mathcal{O}(\chi^7)$; this algorithm is not exact, but its error can be systematically decreased by increasing χ.

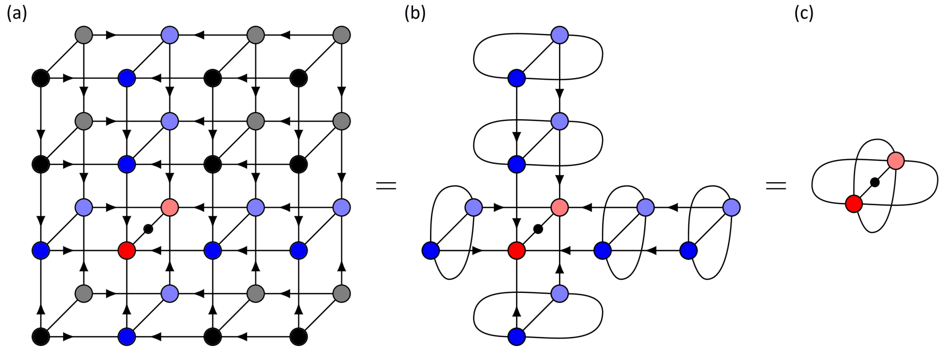

FIG. S11. Evaluation of local observables in an 2D isometric tensor network. (a) Evaluation of $\langle \mathcal{O}_{(2,2)} \rangle$ with site $(2,2)$ being the OC. The expectation value is the double-layer contraction of isoTNS, operator, and conjugated iosTNS. This contraction is greatly simplified using the isometric conditions, first in (b) to the *orthogonality hypersurface*, the generlization of the OC to a 1D object, and then in (c) to the OC itself.

Multi-site operators, such as the weight-10 and weight-17 operators considered experimentally, require a more standard PEPS-style expectation value approach, i.e. contraction of the entire network as shown in Figure S11(a). We can use the isometric conditions on the tensor network to reduce the size of the network to be contracted to just the support of the operator and any tensors in the causal past of these tensors. The causal past is all tensors reached by moving against the arrow direction; so the OC is the earliest tensor causally. We contract the isoTNS just on operator support and its causal cone, the operator, and the conjugate isoTNS to measure the operator. While this can be done with approximate double-layer boundary MPS contractions [S39] with cost $\mathcal{O}(\chi^{10})$, we instead exactly represent the boundary contraction as a dense vector. This approach has no error but incurs an exponential cost in system size; here, system size is defined by the operator support and causal cone isoTNS. We chose this approach to ensure that expectation values of isoTNS are not affected by the bond dimension of the boundary MPS. However, we cannot calculate the expectation value of the weight-17 operator at $\chi = 12$ with this method due to memory constraints.

VII. LIGHT-CONE AND DEPTH REDUCED CIRCUITS

In this section we define the *light-cone and depth reduced* (LCDR) circuits that fascilitate exact simulation and improve the accuracy of approximate simulation. First we discuss how the common structure of the circuits at Trotter depth D and the operators measured in Figures 3 and 4 can be exploited to produce a circuit of reduced Trotter depth $D-1$. Next, we discuss how considering the past lightcone of operators yields a circuit involving fewer gates and qubits. These simplications can be used regardless of the simulation method introduced in VI.

A. Circuit Depth Reduction

We begin by defining a unitary implementing one Trotterized layer of the circuit,

$$U(\theta_h) = \prod_{\langle i,j \rangle} R_{Z_i Z_j}(-\pi/2) \prod_i R_{X_i}(\theta_h) = R_{ZZ}(-\pi/2) R_X(\theta) \tag{4}$$

The state produced by a circuit of depth D is $|\Psi(D,\theta)\rangle = U^D(\theta)\,|0\rangle^{\otimes N}$. Define the operator $Z(D',k)$ as the Pauli string obtained by evolving Z_k by D' layers of the circuit with $\theta_h = \pi/2$; $Z(D',k) = U^{D'}(\pi/2) Z_k U^{D'}(\pi/2)^\dagger$. The three observables considered in the Figure 3 can all be expressed in this way: weight-1 Z_k = Z(0, k), weight-10

stabilizer $Z(5,13)$, and weight-17 stabilizer $Z(5,58)$. The expectation value of $Z(D'>0,k)$ at circuit depth D is

$$\begin{aligned}
\langle Z(D',k)\rangle_{D,\theta} &= \langle\Psi(D,\theta)|\, Z(D',k)\,|\Psi(D,\theta)\rangle \\
&= \langle 0|^{\otimes N}\, U^D(\theta)^\dagger U^{D'}(\pi/2) Z_k U^{D'}(\pi/2)^\dagger U^D(\theta)\,|0\rangle^{\otimes N} \\
&= \langle 0|^{\otimes N}\left[U^{D-1}(\theta)^\dagger R_X(\theta)^\dagger R_{ZZ}(-\pi/2)^\dagger\right] \\
&\quad \left[R_{ZZ}(-\pi/2) R_X(\pi/2) U^{D'-1}(\pi/2) Z_k U^{D'-1}(\pi/2)^\dagger R_X(\pi/2)^\dagger R_{ZZ}(-\pi/2)^\dagger\right] \\
&\quad \left[R_{ZZ}(-\pi/2) R_X(\theta) U^{D-1}(\theta)\right]|0\rangle^{\otimes N} \\
&= \langle\Psi(D-1,\theta)|\, R_X(\theta)^\dagger R_X(\pi/2) Z(D'-1,k) R_X(\pi/2)^\dagger R_X(\theta)\,|\Psi(D-1,\theta)\rangle \\
&= \langle\tilde\Psi(D-1,\theta)|\,\tilde Z(D'-1,k)\,|\tilde\Psi(D-1,\theta)\rangle
\end{aligned} \tag{5}$$

We have defined the modified state $|\tilde\Psi(D-1,\theta)\rangle = R_X(\theta)\,|\Psi(D-1,\theta)\rangle$ and the modified Pauli operator $\tilde Z(D'-1,k) = R_X(\pi/2)Z(D'-1,k)R_X(\pi/2)^\dagger$. So the expectation value of $Z(D',k)$ with circuit depth D can be evaluated using a modified stabilizer at depth $D'-1$ and a modified state with depth $D-1$. Putting aside the change in the operator $Z(D',k) \to \tilde Z(D'-1,k)$ until the next section, requiring a state at one fewer depth provides both computational and experimental benefits; the required bond dimension for tensor network simulations is reduced at depth $D-1$ compared to that at depth D, while experimentally one less layer of two-qubit gates removes a significant source or error in the circuit.

For the weight-1 observable Z_k, note that $R_Z Z(-\pi/2)^\dagger Z_k R_Z Z(-\pi/2) = Z_k$ due to commutation of the gates. Thus, $\langle Z_k\rangle_{D,\theta} = \langle\tilde\Psi(D-1,\theta)|\, Z_k\,|\tilde\Psi(D-1,\theta)\rangle$. So again the expectation value at depth D can be found from the state at depth $D-1$, but now the operator Z_k is unchanged.

Note that the modified weight-17 operator measured in Figure 4(a) is $\tilde Z(5,58)$ with the modified state $|\tilde\Psi(5,\theta)\rangle$. This is equivalent to measuring $Z(6,58)$ with state $|\Psi(6,\theta)\rangle$.

B. Lightcones

Suppose we want to measure $\langle\mathcal{O}\rangle_{D,\theta}$ for a generic operator $\mathcal{O}$. The most straightforward approach, and the one taken experimentally in this work, is to simulate the entire state on N qubits and then measure the operator. However, significant simplications can be made for classical simulations. As the circuit is unitary and local, one could instead simulate only the lightcone of $\mathcal{O}$. For short depth quantum circuits, such as $D=5$ in Figure 3, the lightcone includes a number of qubits $M \ll N$, which makes exact simulations feasible. Additionally, the necessary bond dimension for exact tensor network simulates scales exponentially with system size (e.g. for an MPS, $\chi_{\text{exact}} \leq 2^{L/2}$ for a system of L qubits).

To determine M, consider $\langle\mathcal{O}\rangle_{D,\theta} = \langle 0|^{\otimes N}\, U^D(\theta)^\dagger \mathcal{O} U^D(\theta)\,|0\rangle^{\otimes N}$. M is the size of the support of $\mathcal{O}(D,\theta) = U^D(\theta)^\dagger \mathcal{O} U^D(\theta)$. Single-qubit $R_X(\theta)$ gates do not increase the support. A two-qubit $R_{Z_iZ_j}$ gate grows an operator with support on site i to neighboring site j, as defined by the geometry of the Heavy Hexagon lattice. However, care must be taken when calculating growth in operator support from the R_{ZZ} gates. While one may originally use the three layers of the non-overlapping gates employed to experimentally implement the R_{ZZ} between all nearest-neighbor qubits, since each gate commutes, the support of operator $\mathcal{O}$ grows to include its neighbors upon conjugation by $U^\dagger$. Effectively, we can first conjugate by two-qubit gates that do not act on $\mathcal{O}$ and then act by gates that act nontrivially on the operator. As noted in the inset of Figure 3, the lightcones of weight-1 Z_k, the weight-10 stabilizer, and the weight-17 stabilizers are ≤ 31 (depending on the coordination number and location of Z_k), 37, and 68 qubits, respectively. Using the circuit depth simplification discussed in the previous section, the lightcones can be greatly reduced to ≤ 19, 26, and 48, respectively, as we can ignore a layer of two-qubit gates. In addition to the benefit of one fewer layer of two-qubit gates from depth reduction from $D \to D-1$, for the high weight stabilizers $Z(5,k)$, we also get a reduction in the support of the original operator $\tilde Z(4,k)$ from depth reduction from $D' \to D'-1$. So we get to start with a smaller operator and grow it with fewer layers of two-qubit gates.

Beyond just simulating qubits within the support of the lightcone, one can also only simulate the gates inside of the lightcone; gates outside of the lightcone combine with their Hermitian conjugate in $\langle\mathcal{O}\rangle_{D,\theta}$. Each two-qubit gate grows the bond dimension which requires truncation and thus incurs error. Additionally, as discussed below, the exact bond-dimension for MPS simulations at $\theta_h = \pi/2$ depends on the growth of the support of the initial stabilizers Z_k; each layer of gates grows the stabilizers, so any reduction in the gates applied reduces the needed bond dimension for exact simulation.

VIII. EXACT MATRIX PRODUCT STATES FOR STABILIZER CIRCUITS

Here we discuss how to determine the bond dimension needed to exactly represent a Pauli stabilizer state, a state completely specified by commuting, independent Pauli operators, as an MPS. When $\theta_h = 0$ and $\pi/2$, the circuits we consider in this work are Clifford and thus produce stabilizer states. We use standard stabilizer evolution algorithms [S22, S40] to evolve the initial stabilizer generators into the final ones for the desired depth; e.g. for the initial state $|0\rangle^{\otimes 127}$ with generators Z_i, we evolve these by conjugation by $U(\theta_h)$ defined in Eq. 4. Note that we can do this for light-cone and depth reduced circuits in addition to the full-state circuit. Given a set of stabilizer generators $\mathcal{G}(\mathcal{S})$ for the stabilizer group $\mathcal{S}$ specifying a stabilizer state, the entanglement entropy with respect to a bipartition $(A, \overline{A})$ is given by

$$S_A = N_A - |\mathcal{G}(\mathcal{S}_A)|, \tag{6}$$

where $\mathcal{G}(\mathcal{S}_A)$ is an arbitrary set of generators of $\mathcal{S}_A$, $|\cdot|$ denotes the number of elements in the set, and N_A is the number of qubits in region A [S41, S42]. As Rényi entropies of region A are independent of the Rényi index for stabilizer states, the Schdmit values on the biparition between A and $\overline{A}$ are exactly flat, i.e. a flat entanglement spectrum. Thus given S_A, the Schmidt rank, i.e. number of states that need to be kept in the state decomposition, at bipartition $(A, \overline{A})$ is 2^{S_A}.

To connect this with the bond dimension needed to exactly represent a stabilizer state, note that an MPS is canonical form that can be thought of as a Schmidt decomposition at every possible bipartition along the 1D chain. The rank of Schmidt decomposition determines the bond dimension needed for exact representation. Thus, we can determine the needed bond dimension for exact MPS representation from the stabilizer generators alone that specify the stabilizer state. The bond dimension at each bond of the MPS is simply $\chi = e^S$, where S is the entanglement entropy for that bipartition. We demonstrate this for the 127-qubit circuit at $\theta_h = \pi/2$ as a function of circuit depth in Figure S12. Note that the exact bond dimension for non-Clifford θ_h is not known but is upperbounded by the value for the non-trivial Clifford circuit.

For the experiment in Figure 4(b), magnetization at depth 20 for qubit 62, let us consider the achievable bond dimensions given access to the Summit supercomputer, which as 250 PB of memory. As detailed previously, we use variational compression for MPO-MPS contraction to do time evolution by the two-qubit gates [S24]. This requires the storage of $N = 127$ MPO environments with size $\chi^2 * D$, where χ is the bond dimension of the MPS and D is the bond dimension of the MPO. Additionally, we must store the $N = 127$ tensors that define the MPS, each of size $2 * \chi^2$ (ignoring boundary effects of the first and last tensor). So in total, the required memory, assuming complex entries requiring 16 bytes each, is

$$\text{Memory (Bytes)} = 127 * \chi^2 * 16 * (D + 2) \tag{7}$$

The largest bond dimension of the three MPOs that represent the layer of two-site gates is $D = 16$, so for $\chi = 1024$, our memory estimate is 38 GB (in practice this is an underestimate of the memory usage as additional data is stored during a simulation). Given an allowance of 250 PB, we can achieve a bond dimension of $\chi_{\text{Summit}} = 2.5 \times 10^6$.

To calculate the fidelity of the state with χ_{Summit} with the exact state with $\chi_{\text{exact}} = 72057594037927936$ with the Clifford $\theta_h = \pi/2$ circuit, note that as this state is a stabilizer state, all singular values are equal. For the singular values s_i on a particular bond, we require that $\sum s_i^2 = 1$. Thus for the exact state $s_i^2 = 1/\chi_{\text{exact}}$. The fidelity of the truncated state with the exact state is $\mathcal{F} = |\langle\Psi_{\text{Summit}}|\Psi_{\text{exact}}\rangle|^2$, which when considering truncation at a single bipartition reduces to the total weight of the squared singular values kept in the truncated state. Thus, $\mathcal{F} = \chi_{\text{Summit}}/\chi_{\text{Exact}} = 3.5 \times 10^{-11}$. It is not clear, however, how the fidelity of the truncated state translates to physical expectation values, especially for those of small weight.

IX. DETAILS OF CLASSICAL SIMULATIONS IN FIGURES 3 AND 4

Here we provide details on which specific classical simulation methods were used for each figure in the main text.

A. Figure 3

(a) The exact curve (gray) is found by brute-force simulations of the light-cone and depth reduced circuit. The MPS (pink) and isoTNS (orange) curves are for full-state, 127-qubit simulations of the experimental, i.e. depth 5 and not LCDR, circuit with $\chi = 1024$ and $\chi = 12$, respectively.

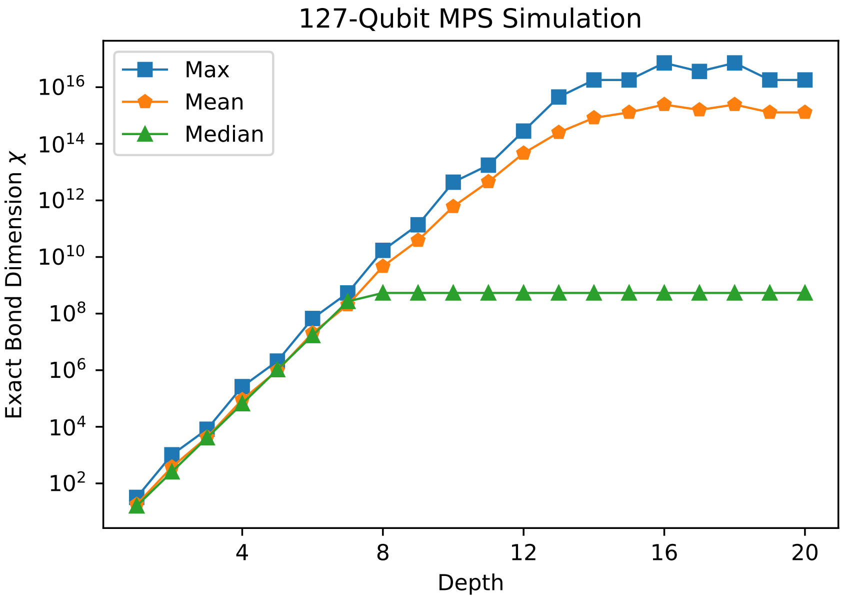

FIG. S12. Max, mean, and median bond dimensions needed to exactly represent state produced by circuit with $\theta_h = \pi/2$ as a function of circuit depth. Max, mean, and median are taken with respect to the 126 bonds in the MPS. 'IBM' ordering is used for the MPS. Bond dimensions are determined by evolving the stabilizer generators Z_i of the initial state $|0\rangle^{\otimes 127}$ and determining the entanglement of each bipartition. Note that the maximum bond dimension required is not monotonic in depth but instead fluctuates when the circuit is deep enough such that all of the initially single-site stabilizers have spread to the entire system.

(b) The exact curve (gray) is found by brute-force simulations of the LCDR circuit of 26 qubits. The MPS (pink) and isoTNS (orange) curves are for full-state, 127-qubit simulations of the experimental, i.e. depth 5 and not LCDR, circuit with $\chi = 1024$ and $\chi = 12$, respectively.

(c) The exact curve (gray) is found by $\chi = 2048$ simulations of the 47-qubit LCDR circuit. This number of qubits is beyond brute-force simulations. The MPS (pink) curve is for full-state, 127-qubit simulations of the experimental, i.e. depth 5 and not LCDR, circuit with $\chi = 1024$. isoTNS results for this operator are not possible with our current expectation value method and memory limitations.

B. Figure 4

Exact simulation is not possible, even using the LCDR circuits.

(a) The MPS (pink) curve is given by 68-qubit LCDR circuit at depth 5 with $\chi = 3072$. The MPS simulations were only evolved for 4 Trotter steps due to depth reduction. isoTNS results for this operator are not possible with our current expectation value method and memory limitations.

(b) The MPS (pink) and isoTNS (orange) curves are given by the 127-qubit, LCDR circuit at Trotter depth 20 with $\chi = 1024$ and 127-qubit, full-circuit simulations at depth 20 with $\chi = 12$, respectively. The bond dimension needed for MPS of the experimental circuit is 18014398509481984, while for the light-cone reduced circuit the bond dimension is 36028797018963968. Although the light-cone reduced circuit excludes gates that exit the lightcone, the lightcone covers the entire chip for 6 layers of two-qubits. We conjecture that the stabilizers have grown sufficiently within these layers that the spatial extent of the operators is not inhibited when the lightcone shrinks at deeper layers. We find that the light-cone reduced circuit produces a stabilizer state that requires larger bond dimension that the original circuit at one larger depth.

X. ADDITIONAL NUMERICAL DATA

A. Magnetization with increasing χ at 20 Trotter steps

In Figure 4(b), we consider Z_{62} at 20 Trotter steps. The lightcone of the operator covers the entire 127-qubit device, and exact simulations are not possible. Instead, we perform MPS simulations of the LCDR with increasing bond dimensions, the results of which are shown below in Figure S13. We find that the measured observable $\langle Z_{62} \rangle$ moves towards the experimental value with increasing χ, but at the maximum value we tried $\chi = 1024$, there are still significant differences between the numerics and the experimental results.

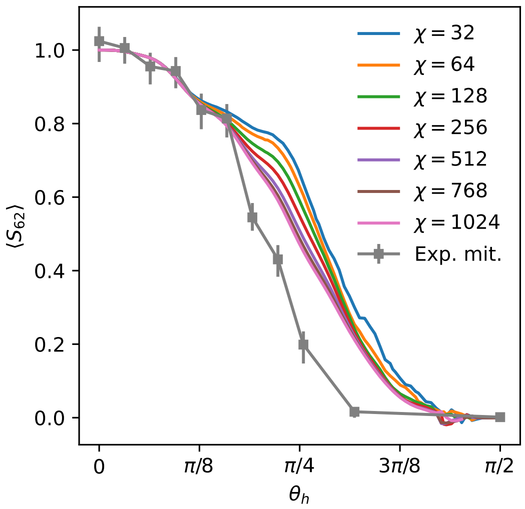

FIG. S13. Depth-20 $\langle Z_{62} \rangle$ using MPS simulations of the LCDR circuit with varying values of χ. The numerical results with increasing bond dimension appear to be moving towards the experimental value.

[S1] Chamberland, C., Zhu, G., Yoder, T. J., Hertzberg, J. B. & Cross, A. W. Topological and subsystem codes on low-degree graphs with flag qubits. *Phys. Rev. X* **10**, 011022 (2020). URL `https://link.aps.org/doi/10.1103/PhysRevX.10.011022`.

[S2] Motzoi, F., Gambetta, J. M., Rebentrost, P. & Wilhelm, F. K. Simple pulses for elimination of leakage in weakly nonlinear qubits. *Phys. Rev. Lett.* **103**, 110501 (2009). URL `https://link.aps.org/doi/10.1103/PhysRevLett.103.110501`.

[S3] Paraoanu, G. Microwave-induced coupling of superconducting qubits. *Physical Review B* **74**, 140504 (2006).

[S4] Chow, J. M. *et al.* Simple all-microwave entangling gate for fixed-frequency superconducting qubits. *Phys. Rev. Lett.* **107**, 080502 (2011). URL `https://link.aps.org/doi/10.1103/PhysRevLett.107.080502`.

[S5] Sheldon, S., Magesan, E., Chow, J. M. & Gambetta, J. M. Procedure for systematically tuning up cross-talk in the cross-resonance gate. *Phys. Rev. A* **93**, 060302 (2016). URL `https://link.aps.org/doi/10.1103/PhysRevA.93.060302`.

[S6] Sundaresan, N. *et al.* Reducing unitary and spectator errors in cross resonance with optimized rotary echoes. *PRX Quantum* **1**, 020318 (2020). URL `https://link.aps.org/doi/10.1103/PRXQuantum.1.020318`.

[S7] Hertzberg, J. B. *et al.* Laser-annealing josephson junctions for yielding scaled-up superconducting quantum processors. *npj Quantum Information* **7**, 129 (2021). URL `https://doi.org/10.1038/s41534-021-00464-5`.

[S8] van den Berg, E., Minev, Z. K. & Temme, K. Model-free readout-error mitigation for quantum expectation values. *Phys. Rev. A* **105**, 032620 (2022). URL `https://link.aps.org/doi/10.1103/PhysRevA.105.032620`.

[S9] Krebsbach, M., Trauzettel, B. & Calzona, A. Optimization of richardson extrapolation for quantum error mitigation (2022). URL `https://arxiv.org/abs/2201.08080`.

[S10] Klimov, P. V. *et al.* Fluctuations of energy-relaxation times in superconducting qubits. *Phys. Rev. Lett.* **121**, 090502 (2018). URL `https://link.aps.org/doi/10.1103/PhysRevLett.121.090502`.

[S11] Carroll, M., Rosenblatt, S., Jurcevic, P., Lauer, I. & Kandala, A. Dynamics of superconducting qubit relaxation times. *npj Quantum Inf* **9**, 132 (2022).

[S12] Runge, C. Über empirische Funktionen und die Interpolation zwischen äquidistanten Ordinaten. *Zeitschrift für Mathematik und Physik* **46**, 224 (1856).

[S13] Temme, K., Bravyi, S. & Gambetta, J. M. Error mitigation for short-depth quantum circuits. *Phys. Rev. Lett.* **119**, 180509 (2017). URL `https://link.aps.org/doi/10.1103/PhysRevLett.119.180509`.

[S14] Quek, Y., França, D. S., Khatri, S., Meyer, J. J. & Eisert, J. Exponentially tighter bounds on limitations of quantum error mitigation. *arXiv preprint arXiv:2210.11505* (2022).

[S15] Tsubouchi, K., Sagawa, T. & Yoshioka, N. Universal cost bound of quantum error mitigation based on quantum estimation theory. *arXiv preprint arXiv:2208.09385* (2022).

[S16] Takagi, R., Endo, S., Minagawa, S. & Gu, M. Fundamental limits of quantum error mitigation. *npj Quantum Information* **8**, 114 (2022). URL https://doi.org/10.1038/s41534-022-00618-z.

[S17] Tran, M. C., Sharma, K. & Temme, K. Locality and error mitigation of quantum circuits (2023). 2303.06496.

[S18] van den Berg, E., Minev, Z. K., Kandala, A. & Temme, K. Probabilistic error cancellation with sparse pauli-lindblad models on noisy quantum processors (2022). URL https://arxiv.org/abs/2201.09866.

[S19] Lisenfeld, J. *et al.* Electric field spectroscopy of material defects in transmon qubits. *npj Quantum Information* **5**, 105 (2019). URL https://doi.org/10.1038/s41534-019-0224-1.

[S20] de Graaf, S. E. *et al.* Two-level systems in superconducting quantum devices due to trapped quasiparticles. *Science Advances* **6**, eabc5055 (2020). URL https://www.science.org/doi/abs/10.1126/sciadv.abc5055. https://www.science.org/doi/pdf/10.1126/sciadv.abc5055.

[S21] Cai, Z. Multi-exponential error extrapolation and combining error mitigation techniques for nisq applications. *npj Quantum Information* **7**, 80 (2021). URL https://doi.org/10.1038/s41534-021-00404-3.

[S22] Gottesman, D. Stabilizer codes and quantum error correction. *Ph. D. Thesis, California Institute of Technology* (1997).

[S23] Cirac, J. I., Pérez-García, D., Schuch, N. & Verstraete, F. Matrix product states and projected entangled pair states: Concepts, symmetries, theorems. *Rev. Mod. Phys.* **93**, 045003 (2021). URL https://link.aps.org/doi/10.1103/RevModPhys.93.045003.

[S24] Paeckel, S. *et al.* Time-evolution methods for matrix-product states. *Annals of Physics* **411**, 167998 (2019). URL https://www.sciencedirect.com/science/article/pii/S0003491619302532.

[S25] Schollwöck, U. The density-matrix renormalization group in the age of matrix product states. *Annals of Physics* **326**, 96–192 (2011). URL https://www.sciencedirect.com/science/article/pii/S0003491610001752. January 2011 Special Issue.

[S26] Hauschild, J. & Pollmann, F. Efficient numerical simulations with tensor networks: Tensor network python (TeNPy). *SciPost Physics Lecture Notes* (2018). URL https://doi.org/10.21468%2Fscipostphyslectnotes.5.

[S27] Vidal, G. Efficient simulation of one-dimensional quantum many-body systems. *Physical Review Letters* **93** (2004). URL https://doi.org/10.1103%2Fphysrevlett.93.040502.

[S28] Zhou, Y., Stoudenmire, E. M. & Waintal, X. What limits the simulation of quantum computers? *Phys. Rev. X* **10**, 041038 (2020). URL https://link.aps.org/doi/10.1103/PhysRevX.10.041038.

[S29] Ayral, T. *et al.* A density-matrix renormalization group algorithm for simulating quantum circuits with a finite fidelity (2022). URL https://arxiv.org/abs/2207.05612.

[S30] Richter, S. *Construction of states on two-dimensional lattices and quantum cellular automata* (Shaker, 1995).

[S31] Niggemann, H., Klümper, A. & Zittartz, J. Quantum phase transition in spin-3/2 systems on the hexagonal lattice—optimum ground state approach. *Zeitschrift für Physik B Condensed Matter* **104**, 103–110 (1997).

[S32] Sierra, G. The density matrix renormalization group, quantum groups and conformal field theory. In *Proceedings of the Workshop on the Exact Renormalization Group* (World Scientific, 1998).

[S33] Nishino, T. & Okunishi, K. A density matrix algorithm for 3d classical models. *Journal of the Physical Society of Japan* **67**, 3066–3072 (1998).

[S34] Verstraete, F. & Cirac, J. I. Renormalization algorithms for quantum-many body systems in two and higher dimensions. *arXiv preprint cond-mat/0407066* (2004).

[S35] Zaletel, M. P. & Pollmann, F. Isometric tensor network states in two dimensions. *Phys. Rev. Lett.* **124**, 037201 (2020). URL https://link.aps.org/doi/10.1103/PhysRevLett.124.037201.

[S36] Lin, S.-H., Zaletel, M. P. & Pollmann, F. Efficient simulation of dynamics in two-dimensional quantum spin systems with isometric tensor networks. *Physical Review B* **106** (2022). URL https://doi.org/10.1103%2Fphysrevb.106.245102.

[S37] Wu, Y., Anand, S., Lin, S.-H., Pollmann, F. & Zaletel, M. P. Two dimensional isometric tensor networks on an infinite strip (2022). URL https://arxiv.org/abs/2211.14337.

[S38] Soejima, T. *et al.* Isometric tensor network representation of string-net liquids. *Physical Review B* **101** (2020). URL https://doi.org/10.1103%2Fphysrevb.101.085117.

[S39] Lubasch, M., Cirac, J. I. & Bañ uls, M.-C. Algorithms for finite projected entangled pair states. *Physical Review B* **90** (2014). URL https://doi.org/10.1103%2Fphysrevb.90.064425.

[S40] Aaronson, S. & Gottesman, D. Improved simulation of stabilizer circuits. *Phys. Rev. A* **70**, 052328 (2004). URL https://link.aps.org/doi/10.1103/PhysRevA.70.052328.

[S41] Hamma, A., Ionicioiu, R. & Zanardi, P. Bipartite entanglement and entropic boundary law in lattice spin systems. *Physical Review A* **71** (2005). URL https://doi.org/10.1103%2Fphysreva.71.022315.

[S42] Nahum, A., Ruhman, J., Vijay, S. & Haah, J. Quantum entanglement growth under random unitary dynamics. *Physical Review X* **7** (2017). URL https://doi.org/10.1103%2Fphysrevx.7.031016.
